# Augmenting geovisual analytics of social media data with heterogeneous information network mining—Cognitive plausibility assessment

**DOI:** 10.1371/journal.pone.0206906

**Published:** 2018-12-04

**Authors:** Alexander Savelyev, Alan M. MacEachren

**Affiliations:** 1 Department of Geography, Texas State University, San Marcos, Texas, United States of America; 2 The Pennsylvania State University, University Park, Pennsylvania, United States of America; GGS, UNITED STATES

## Abstract

This paper investigates the feasibility, from a user perspective, of integrating a heterogeneous information network mining (HINM) technique into SensePlace3 (SP3), a web-based geovisual analytics environment. The core contribution of this paper is a user study that determines whether an analyst with minimal background can comprehend the network data modeling metaphors employed by the resulting system, whether they can employ said metaphors to explore spatial data, and whether they can interpret the results of such spatial analysis correctly. This study confirms that all of the above is, indeed, possible, and provides empirical evidence about the importance of a hands-on tutorial and a graphical approach to explaining data modeling metaphors in the successful adoption of advanced data mining techniques. Analysis of outcomes of data exploration by the study participants also demonstrates the kinds of insights that a visual interface to HINM can enable. A second contribution is a *realistic case study* that demonstrates that our HINM approach (made accessible through a visual interface that provides immediate visual feedback for user queries), produces a clear and a positive difference in the outcome of spatial analysis. Although this study does not aim to validate HINM as a data modeling approach (there is considerable evidence for this in existing literature), the results of the case study suggest that HINM holds promise in the (geo)visual analytics domain as well, particularly when integrated into geovisual analytics applications. A third contribution is a *user study protocol* that is based on and improves upon the current methodological state of the art. This protocol includes a hands-on tutorial and a set of realistic data analysis tasks. Detailed evaluation protocols are rare in geovisual analytics (and in visual analytics more broadly), with most studies reviewed in this paper failing to provide sufficient details for study replication or comparison work.

## 1. Introduction

A primary objective of the developing field of geovisual analytics is to support analytical reasoning and sense-making with large, heterogeneous data that include reference to place. An example of a software system designed to support this objective is SensePlace3 (SP3), a multi-year project that aims to combine interactive visual interfaces with computational modeling techniques to help the analyst achieve insight about geospatial social media data [1]. We have recently introduced a new analytical extension to the SP3 project that employs network modelling techniques to make sense of large collections of *interconnected entities* (hashtags, place mentions, etc.) inherent in social media data. Specifically, our extension makes use of a Heterogeneous Information Network Mining approach (HINM), a recently introduced network-centric computational technique [2]. HINM leverages connections across heterogeneous data to support complex queries. It matches the existing data model of SP3 closely and has been documented to be a success for uncovering patterns, which might otherwise remain hidden, across a number of application areas, making it a promising candidate for inclusion into the SP3 toolset.

The key purpose of this study, however, is not to evaluate the HINM as a data modelling approach, as this was already done elsewhere [3–5], but to assess its understandability and utility in realistic geospatial analytical scenarios. Specifically, it is the complexity of the data modeling metaphors employed by HINM that represents a significant adoption challenge. The notion of the heterogeneous networks, the use of non-intuitive concepts to establish links between elements of said network, the complexity of the algorithms involved in data analysis as well as the lack of obvious visual metaphors for use in a (geo)visual workflow has thus far restricted this technique to the data mining community with significant technical background and data mining expertise. Although cognitive challenges associated with network visualization in general have been explored before (e.g. [6, 7]), there is no current work on the cognitive and perceptual issues associated with interpretation and understanding of HINM-based tools, specifically, and it is common for existing visual analytics work to either focus on homogenous network analysis processes (e.g. [8, 9]), or on the utility of a tool rather than its usability (e.g. [10–13]). In order to explore the feasibility of using the HINM technique in the framework of geovisual analytics, we have:

1. Created an extension component to the SP3 application that integrates key elements of the HINM approach with the geovisual analytics workflow.
2. Designed and administered a carefully-planned evaluation study that incorporates user training, realistic data exploration and analysis, and comprehension evaluation.

Both of these steps are described in detail below.

## 2. Study background

SensePlace3 is a web-based geovisual analytics environment developed to improve situational awareness in the domain of crisis management and the related fields [1]. We are currently using Twitter as the principal source of geospatial information, although the SP3 system itself is capable of ingesting text data from other sources as well. As part of the data processing step, SP3 extracts a number of *linked entities* from the original tweets. Specific types of entities include snippets of Twitter metadata (e.g. message timestamps, GPS coordinates, hashtags mentions, re-tweets and @mentions of Twitter users) as well as *place mentions*. The place mentions are further processed with GeoTxt [14], a geoparsing API that extracts and geocodes references to places contained in the unstructured text of the individual tweets. Overall, the key aim of SP3 is to provide the analyst with a set of interactive geovisual analytics tools specifically tailored to support flexible exploration and analysis of the multi-dimensional spatio-temporal datasets.

### 2.1 SensePlace3 overview

**Fig 1:**
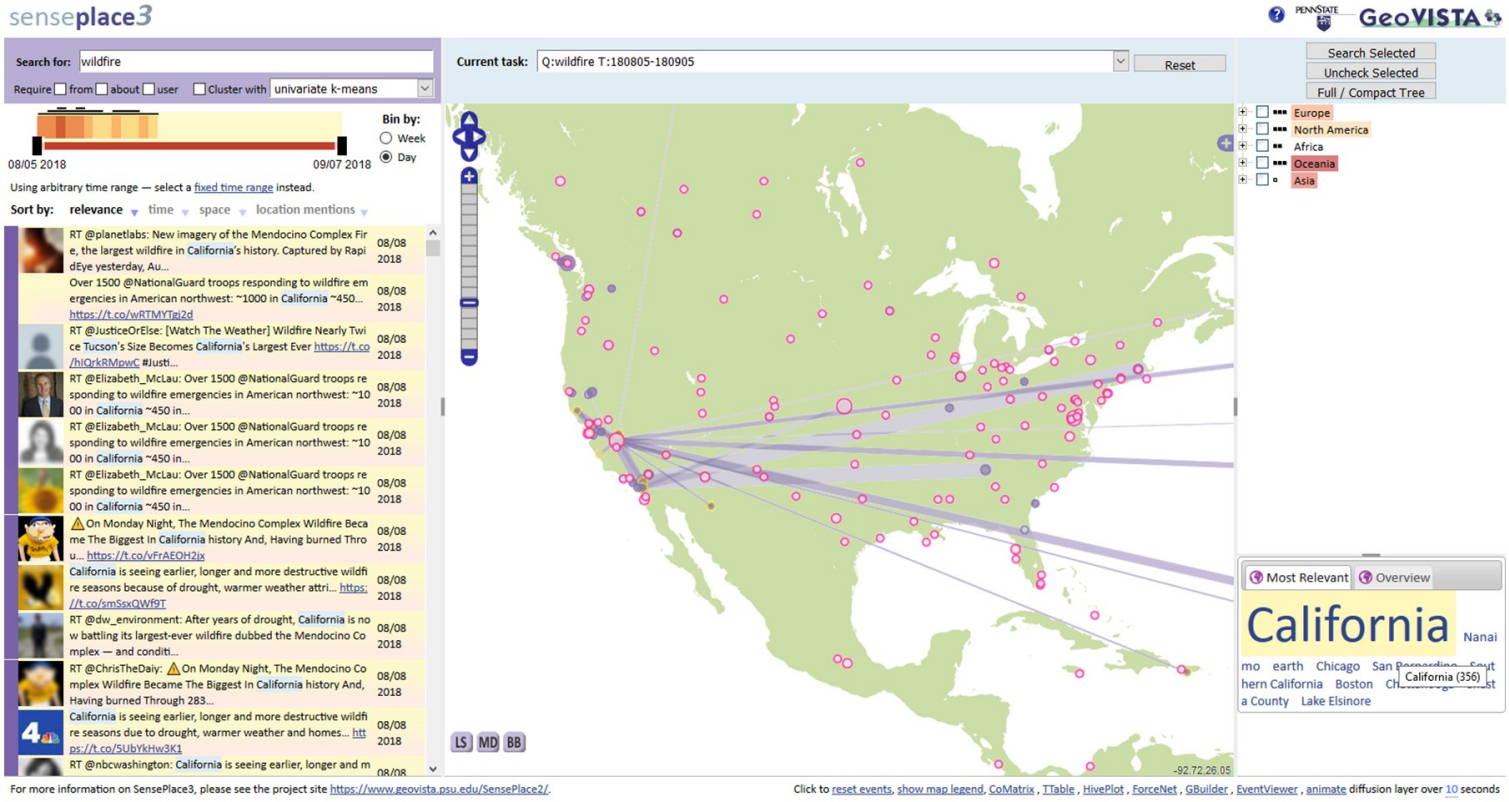
Main window interface of the SensePlace3. This example shows a query on “wildfire” in August, 2018 after serious wildfires resulted in many deaths and destroyed thousands of homes in California. The highlighted tweets were picked by clicking on places and show a few of the postings at the height of the fires. Connection lines show links among places and “California” based on co-mention in the relevant tweets. This figure is for illustrative purposes only–the actual SP3 makes use of Google Maps for the base layer, which was replaced by a generic outline of the US (available under CC BY 4.0 license at https://commons.wikimedia.org/wiki/File:World_map_(Mercator_projection)_Pacific-centric.svg) for copyright reasons.

SensePlace3 is designed as a *coordinated collection* of individual tools that enable users to explore and analyze different dimensions of social media data, including spatial, temporal and thematic dimensions. Fig 1 provides an overview of the SP3 main window interface.

In the upper-left corner of Fig 1 a *search box* is visible, with a single keyword–“wildfire”–used to generate this example. Below the search box is an *interactive timeline* showing temporal trends in the available data that matches the keyword query for a one-month window starting from August 5^th^ of 2018. A *tweet list–*a list of the 1,000 most relevant tweets retrieved in response to the query appears below the timeline. To the right of this panel, the time and attribute views are complemented by three spatial views that provide different perspectives on place references extracted from the tweets: *map* (center), *hierarchical place tree* (upper right) and the *place mention tag cloud* (lower right). The hierarchical place tree and the place mention tag cloud both focus on place mentions in tweet text, while the map depicts these data along with the “from” location for any tweet that is geolocated and the “profile” location for any tweet with a recognizable place in the tweeter profile.

All components in SP3 interact with each other, with individual components linked together using a coordinated view approach that adheres to the “overview first, zoom and filter, then details-on-demand” mantra [15]. As seen in Fig 1, several locations have been clicked on in the map view, causing the place label associated with them to display. Following each click, the individual tweets that mention, are issued from, or have a particular profile location are sorted to the top of the tweet list and are highlighted in color as well (left side of the figure). When the mouse moves over the top tweet in the list, which mentions “California”, links are displayed on the map between California and any other place mentioned in a tweet that also mentions California (from the top 1000 currently in the client). This and other interactions between the SP3 components follow a cross-filtered view design pattern, implemented using a JavaScript visual coordination library reported on previously [16]. A comprehensive discussion of the architecture and full functionality of the SP3 interface (omitted here for brevity) is available elsewhere [1]. Here we focus on the connections between data components and how a visual analytics interface implementing an HINM approach can help users leverage those connections.

### 2.2 Exploring connections in multi-dimensional geospatial datasets

Most of the SP3 tools (e.g. map, timeline, and tweet list) are tailored to specific dimensions of the SP3 dataset and allow for in-depth analysis and exploration of said dimensions, but this approach only works for a small *subset* of the data variables. The full set of available variables is too large to analyze using “one component per variable” approach, and an alternative solution is needed. Moreover, the coordinated view approach, illustrated by the map click example in the previous section, is quite flexible, but is aimed at exploring connections between just a few entities at a time, and a different solution is required for generating an *overview* of the multitude of hidden connections that exist in the dataset.

**Fig 2:**
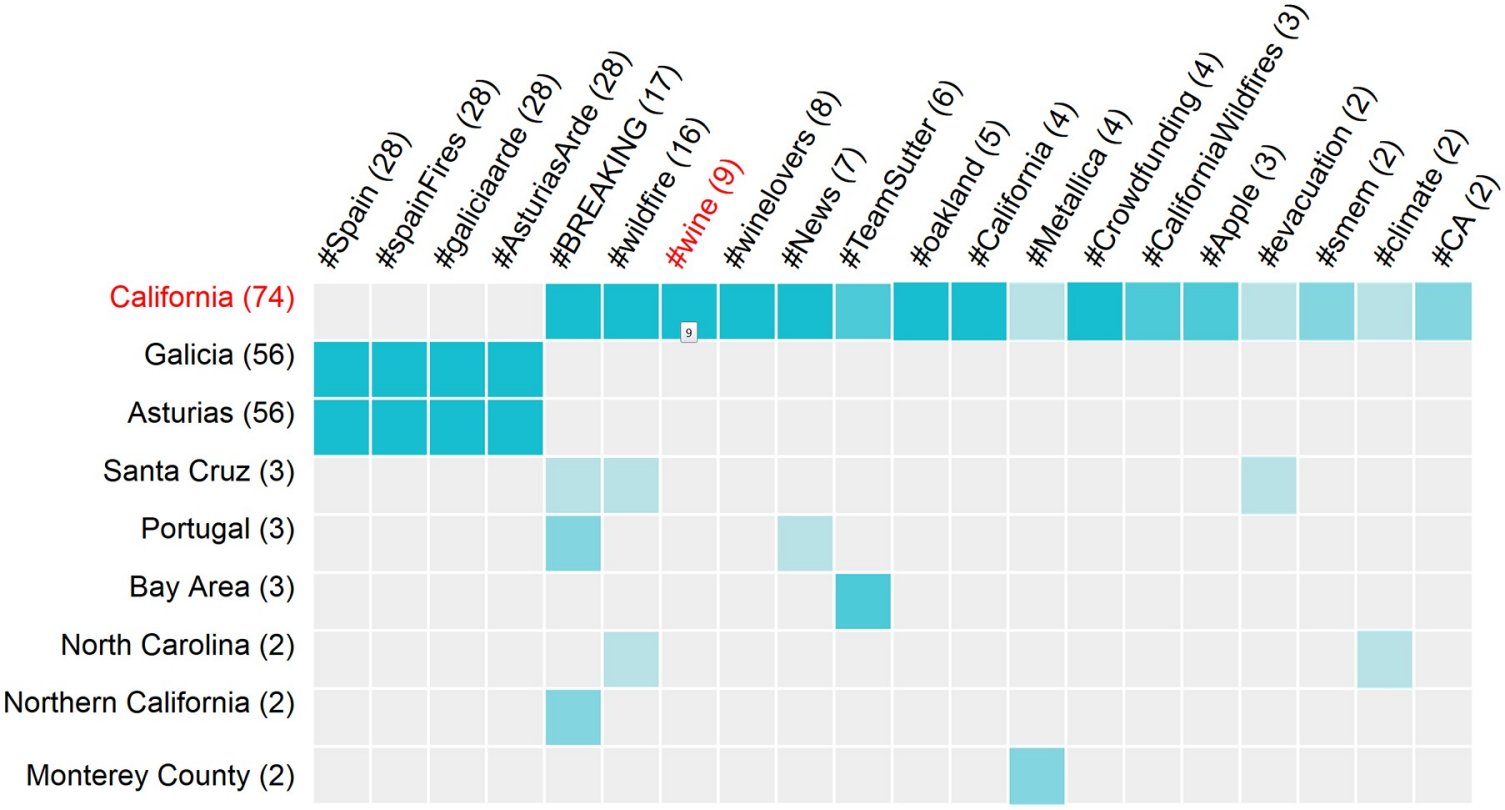
A snippet of the Co-Occurrence Matrix display. This display is generated for the wildfire query for the August of 2018, showing connections between place mentions and hashtags. The cell corresponding to the intersection of *#wine* hashtag and *California* place mention is highlighted, and the number of tweets that simultaneously refer to both of these entities is shown (a total of 9).

An SP3 tool designed specifically to address this challenge is a Co-Occurrence Matrix (*CoMatrix* for short), a snippet of which is shown in Fig 2.

The CoMatrix allows the analyst to plot any two types of the SP3 entities as rows and columns in a re-orderable matrix display [17], with strength of association between individual row and column elements shown as the intensity of the color fill in the corresponding matrix cell. The example shown in Fig 2 displays the association between *place mention* and *hashtag* entities, as identified in the results of the sample query for “wildfire”. A clear association between the place mention of “California” and hashtags related to wine is obvious in this example; the wildfires were most devastating in the wine region of California. CoMatrix is presented to the user as a pop-up window that can be repositioned freely (given its considerable size, it’s hard to integrate it as just another component of main interface), but is dynamically linked to the rest of the SP3 UI using the same “overview first, zoom and filter, then details-on-demand” and coordinated view principles (the mechanism for this cross-window coordination is discussed in detail elsewhere [16]). For example, a click on a cell highlighted in the figure would cause the tweets that mention both of these entities to sort to the top of the tweet list in the primary window and will highlight them in color.

**Fig 3:**
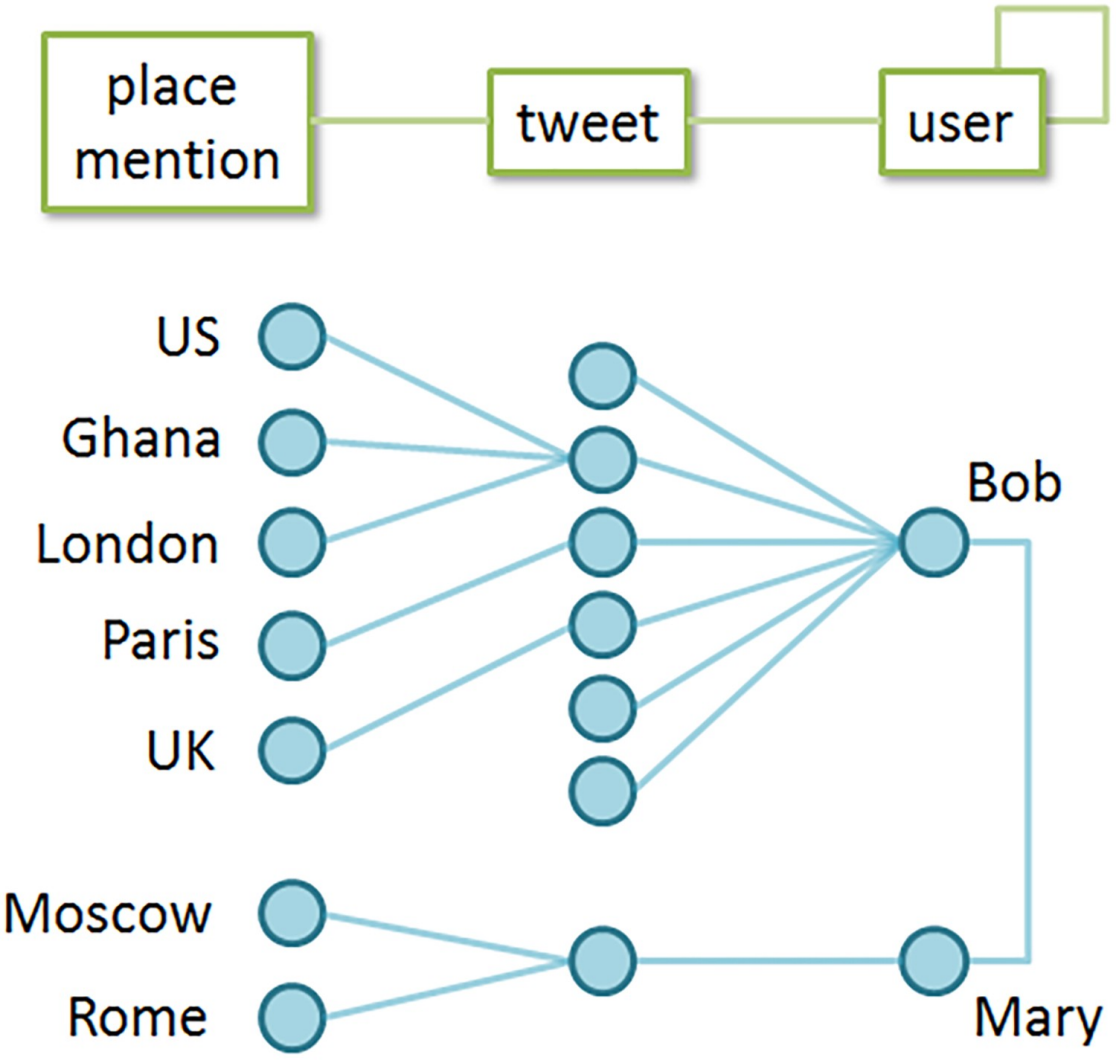
Sample SP3 dataset visualized as a network of connected entities. Entity types and possible connections between them are shown in green, a sample dataset composed of these entity types is shown in blue.

A key challenge we had to address while working on the CoMatrix component is the ambiguity of what constitutes an association between two specific entities in the dataset. Fig 3 illustrates this problem using a small, synthetic dataset with just a few entities present.

The example shown in Fig 3 only makes use of three entity types (place mention, tweet, and user) for the sake of brevity. The upper part of Fig 3 (green) shows types of connections that exist in this dataset–users can author tweets, tweets can contain place mentions, and users can follow each other. The lower part of Fig 3 (blue) shows the actual sample dataset visualized as a network of connected entities, with two users (Bob and Mary) who follow each other on Twitter and write a series of tweets that mention specific places following world-wide news events.

Fig 3 illustrates the ambiguity of what constitutes association between entities in such networks. For example, given connections shown in Fig 3, what are the place mentions associated with “Ghana”? A plausible answer is “US and London”, as these three locations are mentioned in the same tweet. However, other answers are equally plausible. Instead of asking a fairly narrow question ‘what places are mentioned in tweets that mention Ghana’, we can ask a broader question such as ‘if a person mentions Ghana on Twitter, what other places have they talked about’. In this case, the answer will be “US, London, Paris and UK”, as Bob, the author of the tweet about Ghana, mentions all of them in his earlier tweets. Another alternative would be to ask a question ‘if a person mentions Ghana on Twitter, what other places have they and their friends talked about’. Given that Bob is friends with Mary, the answer will include US, London, Paris and UK, and also add Moscow and Rome. All three examples represent valid, yet different, forms of association between place mentions.

This and similar issues had surfaced across multiple domains before, and the problem of sense-making in large, heterogeneous networks is considered a formidable challenge in the literature [18]. Previous work also highlighted the disproportionate amount of focus that binary, low-order relationships receive in network analysis systems (in contrast with more complex, composite relationships described in the previous paragraph) [2]. Overall, there is a growing realization that a flexible tool aimed at exploring high-level, situated relationships is needed [19]. As indicated in the introduction, to augment our visual approach to this problem, we have borrowed a number of metaphors and computational techniques from the domain of Heterogeneous Information Network Mining (HINM). The resulting strategy provides a method to formalize and automate the process of defining, calculating the associations between, and displaying associated entities present in our dataset.

### 2.3 Using heterogeneous information network mining (HINM) metaphors for spatial data analysis

**Fig 4:**
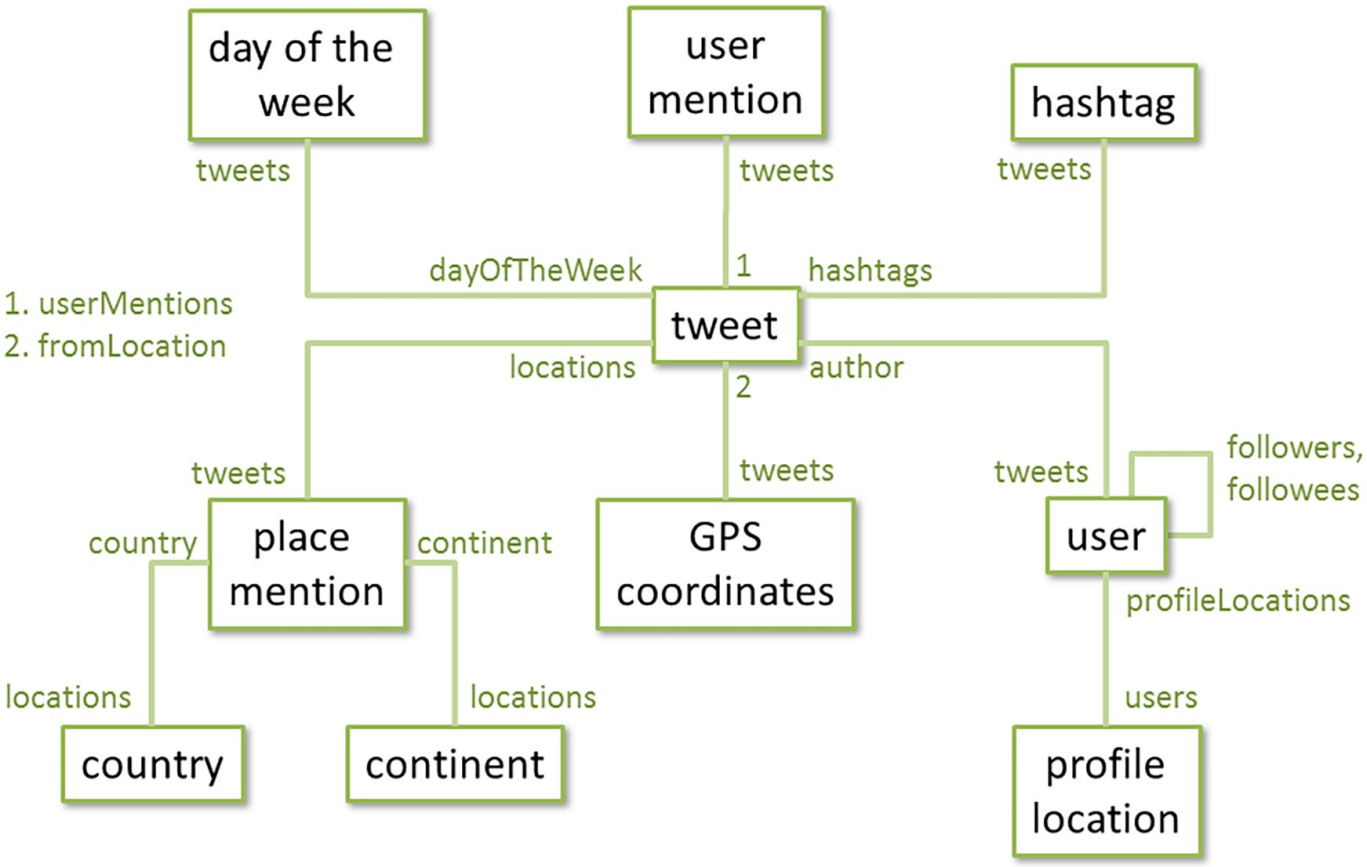
Complete network schema used in the SP3 application. Black labels represent entity types (nodes), green labels represent possible connections between entities (links). Links are labelled at the source, e.g. a link named “locations” (center of figure) can be used to traverse from a particular tweet to all the places it mentions.

Key concepts used in the HINM approach are the notion of the heterogeneous network itself, the notion of a network schema, and the notion of a meta-path [20–22]. *Heterogeneous networks* are networks composed of entities (nodes) and connections (links) between them, where both nodes and links can come in a variety of different types. *Network schema* represent the connections that are possible in a given heterogeneous network. *Meta-path*, described in more detail below, is a formal way of encoding (multi-step) connections between entities in a heterogeneous network. Fig 4 shows the complete network schema used in the current SP3 application.

A network schema is used to define the last key concept of the HINM approach–the meta-path. A *meta-path* can be thought of as the prescribed set of directions for getting across the network schema from one entity type to another. For example, looking previously at Fig 3, we were able to generate three different ways of building associations between place mentions–through their co-occurrence in the same tweet, in the Twitter feed of a particular user, or in a community of users. These three approaches to building associations between place mentions can be formalized as three distinct meta-paths:

*place mention → tweet → place mention*,
*place mention → tweet → user → tweet → place mention*, and
*place mention → tweet → user → user → tweet → place mention*.

### 2.4 Integration of HINM component into a geovisual analytics workflow

We have implemented a prototype HINM tool and integrated its functionality with the Co-Occurrence Matrix component and the SP3 coordination mechanism. This section discusses some of the technical details of this process that were not covered earlier:

1. The web-based heterogeneous network model implementation, and
2. The meta-path selection tool.

#### 2.4.1 Heterogeneous information network models in web-based environments

SensePlace3 takes advantage of JavaScript programming language features, namely mutable objects, to implement its heterogeneous information network model. Individual nodes on the network are represented as JavaScript objects, and store their ‘personal data’ (e.g. node type, its Universal Resource Identifier (URI), etc.) as named properties on same objects. For example, given a node *sampleNode*, one would examine its type by inspecting its property *sampleNode*.*type*.

Links are represented through nodes (JavaScript objects) storing references to each other in an organized fashion. More specifically, some of the object’s properties (its *linked properties*) take the form of an array of references to other objects. Linked properties are also named, which allows the system to establish directed, labelled connections that are the key characteristic of the heterogeneous information network model. For example, given a user node *sampleUser*, one would examine the links to the tweets that user sent by inspecting its property *sampleUser*.*tweets*. Inversely, given a tweet node *sampleTweet*, one would examine the link to its author by inspecting its property *sampleTweet*.*author*. Linked properties are named according to the schema for the heterogeneous network data model shown in Fig 4.

The heterogeneous data model itselfis populated in two steps. First, the query results returned by the SensePlace3 server are parsed to create a list of nodes that will comprise the future network. Most named properties of the nodes (e.g. node type) are set to their final values at this point. The linked properties (i.e. arrays of references to other objects), however, are only set to hold the Universal Resource Identifier (URI) of the linked objects, not the references to the objects themselves. For example, a sample tweet node would have its linked property *author* set to a text string *“user_87746492”* instead of the actual reference to the user node designated by that URI. The second step consists of a second pass through a list of objects where the URIs in the linked properties are replaced with references to real JavaScript objects, thus completing the network. The pseudo-code for this process is as follows:

For each tweet in the current query results,

 for each node type in the network schema,

  construct the network node,

  set its linked properties equal to the URIs of connected nodes,

  set non-linked properties to their values,

  add this node to global list of nodes.

For each node in the global list of nodes,

 for each linked property of the current node,

  look up the corresponding node by the URI stored in the linked property,

  replace the URI with a link to the actual node in the global list.

The reason for the two-step process of building the network is two-fold. First, some of the linked properties (e.g. follower-followee) can be cyclical, which can result in a deadlock situation, when completion of object A requires the instantiation of object B that, in turn, has a reference to object A. Having a two-pass algorithm eliminates this problem completely. Second, a network with linked properties based on URIs allows some objects to not be instantiated during the network creation process, making it possible to build small networks with links to more data on the server, and have that data requested on demand. The ultimate goal of using URIs in this fashion is to allow the system to explore much larger datasets than would be possible otherwise.

The average size of the HINM networks built by the web-based SP3 application is approximately 6,000 nodes and 14,000 links per each 1,000 tweets. We did not encounter any performance issues with the network of this size, and it was primarily limited by the number of tweets it is meaningful to display to the analyst for inspection.

#### 2.4.2 Meta-path selection tool

**Fig 5:**
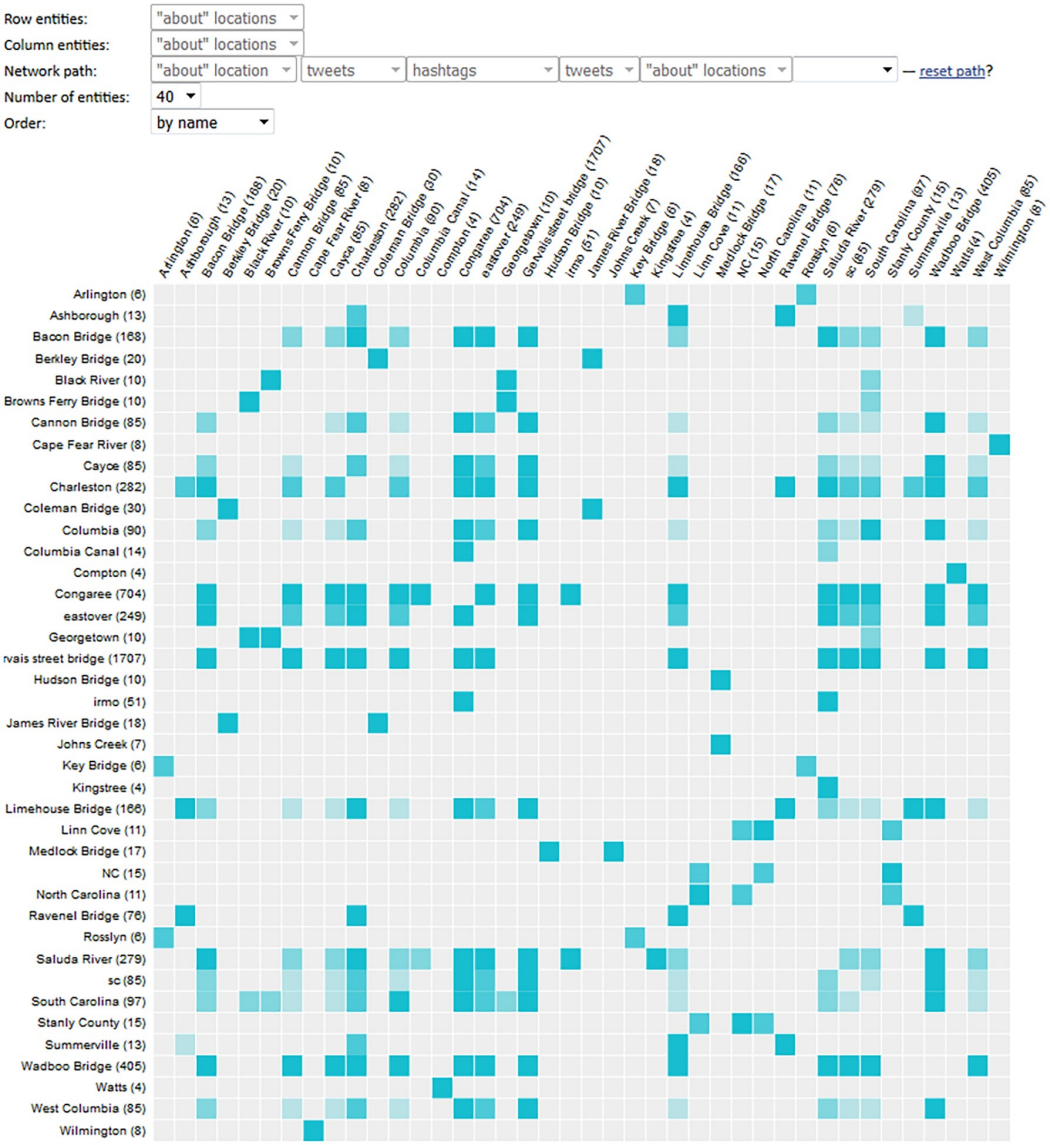
Sample CoMatrix display with the prototype meta-path selection tool. Shown as seen during the study, with the selected *place mention–tweet–hashtag–tweet–place mention* meta-path visible at the top of the figure against the “Network path” label. Meta-paths are constructed by selecting desired entity types (network nodes) in a sequence of drop-down menus. Meta-path length is not limited, with a “blank” drop-down menu automatically added every time a selection is made.

To make our implementation of the HINM approach analytically useful, we have built a user interface tool that enables an analyst to define arbitrary meta-paths through the network of SP3 entities. The prototype meta-path selection tool is demonstrated in Fig 5.

The network path currently selected in Fig 5 is “*place mention–tweet–hashtag–tweet–place mention*”, corresponding to places related by virtue of being mentioned along with the same hashtag. The meta-path selection tool works by offering the analyst a blank drop-box menu that contains the list of all entity types in the network schema. As the analyst makes their first choice (with the place mention, labelled as ‘about’ location, being the first pick in Fig 5), another drop-box is added to the meta-path string. This time, the analyst’s choice will be limited to the entity types that are connected to the place mention in the network schema. By making their pick in the blank drop-down boxes, the analyst is essentially ‘walking’ along the links defined in the network schema. The CoMatrix visualization is dynamically redrawn every time a choice is made, showing the strength of connection between the first and the last item in the selected meta-path, with cells colored proportionately to the total number of connections detected between each specific pair of entities. In the example shown in Fig 5, the analyst made 5 drop-down menu choices so far, is exploring the strength of connection between place mentions, and is offered another drop-down menu in case they intend to continue building the meta-path.

The actual calculation of the strength of connection between the first and the last item in the selected meta-path is performed using an implementation of a depth-first search on the SP3 network model. Although our implementation of the HINM is primarily tailored to the needs of our evaluation study, we took care to implement the co-occurrence detection algorithm as a generic recursive function, following the vertex-centric computation paradigm [23, 24] designed to accommodate distributed computation tasks on very large networks. The rationale behind this decision is similar to that for our use of URIs–future versions of SP3 would shift the network computations from the client to the server, enabling us to explore much larger datasets, with the current implementation serving as a technology demonstrator and a prototype.

## 3. User study design

The goal of this study is to explore whether analysts can comprehend the data modelling metaphors employed by HINM, whether they can employ HINM to explore spatial data, and whether they can comprehend the results of HINM-based analysis. In order to achieve this goal, we first added a visual HINM component to SP3. This section provides details about the user study that we designed and have administered in order to evaluate whether HINM can be understood and used by analysts in a practical data analysis scenario. This user study consists of three individual components:

1. A hands-on, interactive HINM tutorial,
2. Four realistic spatial data analysis tasks that employ HINM-based tools, and
3. A comprehension evaluation task that checks whether participants understand the principles behind the HINM-based spatial analysis.

The spatial data analysis tasks mentioned in item 2 above were also designed in such way that specific findings obtained by participants form a *case study* that demonstrates the applicability and utility of HINM-based geovisualization tools to the problems of spatial data analysis.

Throughout the study, we use short answers to collect data, including participants’ insights for specific analytical tasks, feedback on actual software tools, and to check user comprehension of the HINM concepts. Our data analysis methods follow the user study design suggestions we were able to locate in comparable evaluation studies of geovisual analytics tools, which are: (a) to include verbatim quotations that highlight the most salient findings [25–31]; (b) to generate a digest that provides an abstracted overview of feedback [32–34]; (c) to include task completion rates (in terms of either time or correctness) [34, 35]; and/or (d) to develop and apply an explicit approach to coding free-form participant feedback based upon grounded theory [36].

We also use the NASA Task Load Index (TLI) semantic differential scale [37], a well-established workload scale, to collect participants’ perception of their performance and the effort level required to achieve said performance. NASA TLI uses 6 subscales to evaluate the task workload–perceived mental, physical, and temporal demand, perceived performance, effort and frustration. *Mental demand* corresponds to the “amount of thinking, remembering, and searching”, *physical demand* corresponds to the “amount of clicking, scrolling, and typing”, *temporal demand* corresponds to the amount of time pressure experienced, *performance level* corresponds to the self-assessed level of success with a given task, *effort* captures the amount of work placed into the task to achieve said level of performance, and the last subscale captures the amount of *frustration* experienced during the task [37]. With the exception of the performance subscale, lower scores indicate lower workload, which is often desired. Low performance score, on the other hand, implies a perception of low success rate at accomplishing the task at hand–an undesirable outcome. NASA TLI has been used and validated across a wide range of domains, including visual analytics [28]. Its focus on experienced workload (as compared to, for example, usability, measured by other popular evaluation scales) is a convenient way to assess potential for adoption in real analytical scenarios, given that

“integration of social media analysis in operational activities was considered impractical by practitioners, […] mainly due to the intense workload associated to social media analysis [and] their lack of human resources to dedicate to this task”

[29].

We recruited a total of 11 participants for this study using a combination of message boards, mass emails and paper flyers over a period of three weeks in July of 2016. All of the participants who inquired about the study completed it fully, with the drop-out rate of zero. Participants were offered $20 in cash in exchange for an hour of their time, and had to be over 18 years old and fluent in English. The resulting participant pool included university staff, undergraduate and graduate students, with 7 females and 4 males ranging from young adults to retirement age (as estimated by the experimenter—no age or gender data was collected). Five of the participants who responded to advertisement were students from within the Department of Geography, although none had previous exposure to the HINM-based analytical techniques or their implementation in the SP3 toolkit. The Institutional Review Board (IRB) Program at the Office for Research Protections at The Pennsylvania State University determined that this study "does not require formal IRB review because the research met the criteria for exempt research according to the policies of this institution and the provisions of applicable federal regulations". Participants have provided their consent for the study verbally, after having reviewed the informed consent form, as approved by the Pennsylvania State University IRB.

Participants took an average of 50 minutes to complete the study, with roughly 15 of those 50 minutes spent on the tutorial component. Individual study completion times varied from 30 to 60 minutes. The first two participants used a slightly different version of the study questionnaire, which had a single input field to collect the answers to two related questions. Separate input fields were used for each of the said questions for the third and subsequent participants to avoid possible confusion (see sections 2.3 and 4.3 for a complete summary).

**Fig 6:**
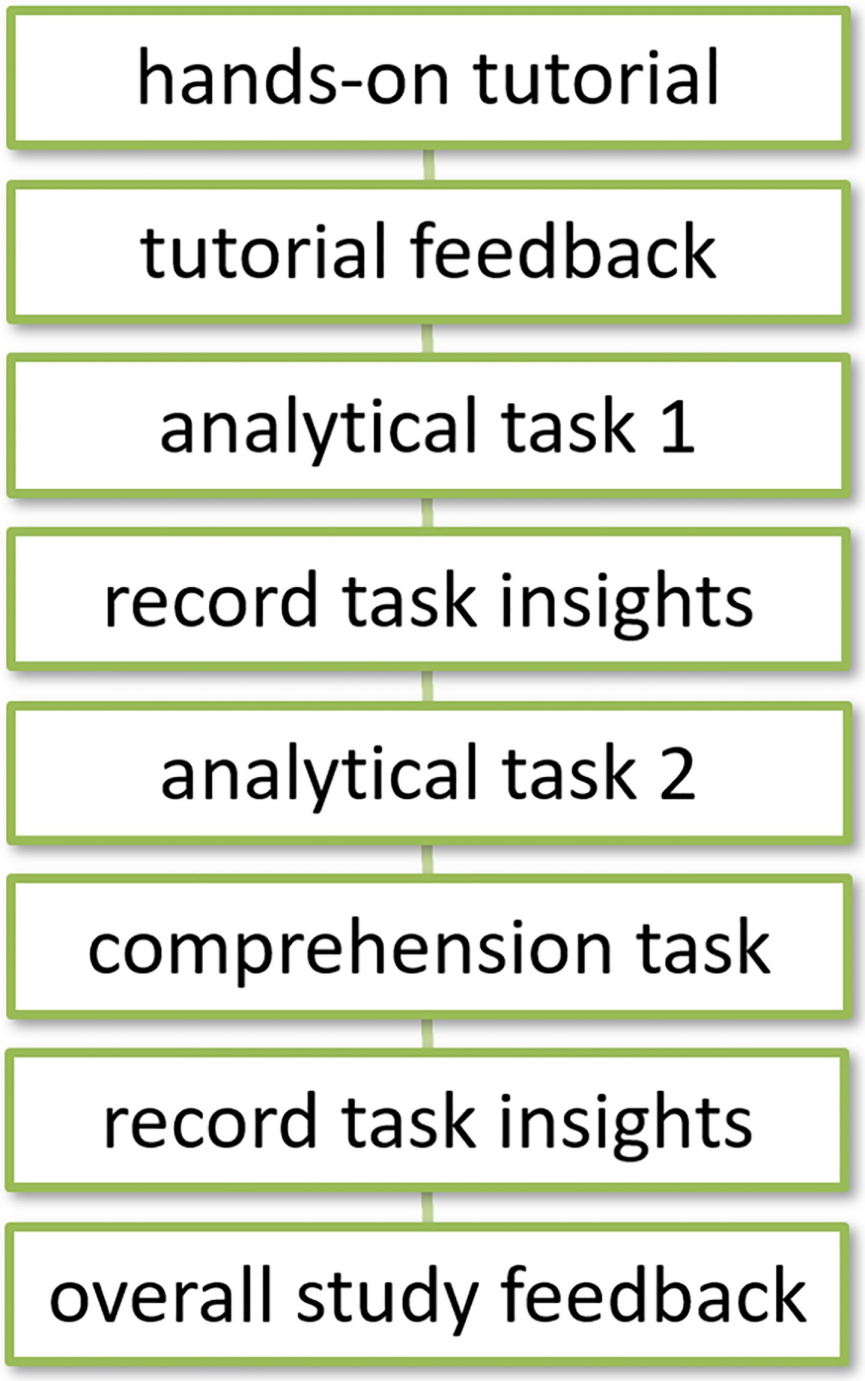
The overall user study workflow.

The overall study workflow is illustrated in Fig 6 below. The following subsections provide further details on the components of this study, with the actual survey instruments provided in the appendix.

### 3.1 Heterogeneous network modelling tutorial

The first component of our user study is a short (15 minutes), hands-on, interactive HINM tutorial designed to explain the basics of the HINM-based visual analysis to study participants. It is generally acknowledged that geovisual analytics tools are complex and carry with them a steep learning curve that will impact the performance of study participants [38–40]. A typical solution to this problem observed in the literature is to provide various forms of instruction and training to the participants, including system overviews, tutorials, walk-throughs, cheat sheets, “controlled” tasks, etc. [25, 28, 30, 31, 33, 35, 36].

An alternative approach is to utilize best domain practices and actively design the system to be less complex in nature and more accessible to study participants without specialized training [27, 29, 34]. As we were not able to locate existing work that integrates HINM with the workflow of geovisual analytics (thus there is no succinct list of best practices for doing so), and we opted for the tutorial approach instead.

The actual tutorial consisted of two parts. First, participants were presented with a number of synthetic tweets (e.g. "Heavy rain in Westmoreland cnty #paWeather #rain") and were asked to convert them into diagrams using pen and paper (examples of diagrams for this and further steps are provided in the appendix).

For the next step, participants were asked to convert the diagrams they just created into a miniature Co-Occurrence Matrix visualization, filling its cells with ink if they corresponded to at least one of the network diagrams built earlier.

In total, participants were asked to manually create two co-occurrence network visualizations corresponding to two different meta-paths, with the first one corresponding to the *hashtag–tweet–hashtag* meta-path and the second one corresponding to the *hashtag–tweet–place mention–tweet–hashtag* meta-path, respectively. The first meta-path will mark hashtags as related if they were mentioned in the same tweet, the second will mark them as related if they were used in a tweet describing a shared location. We did *not* introduce the concept of the meta-path to the study participants, instead referring to example diagrams (provided in the appendix) as two equally plausible ways to mark hashtags as related to each other. These diagrams were also purposefully oriented in a way that places hashtags along the margins in a layout that is similar to their positioning in the co- matrix to make the connection between the network-like diagrams and the CoMatrix explicit.

The tutorial was administered as follows. First, participants were given a chance to read through the description of the current task. Then, the researcher (first author) would volunteer to convert the first synthetic tweet into a network-like diagram (or fill one cell of the CoMatrix based on the first diagram). Finally, participants were asked to convert the rest of the tweets into diagrams, with the researcher checking for correctness of the final result and offering additional explanations, if needed. Prior to proceeding to the next component of the user study, participants were asked to fill out the segment of the questionnaire corresponding to the tutorial component, consisting of a NASA TLI questionnaire and a short-answer section describing thoughts and comments (if any) about the tutorial.

### 3.2 HINM-based spatial analysis tasks

The goal of our investigation–exploring whether analysts can learn, use and comprehend the data modelling metaphors employed by HINM in a practical data analysis setting–is modelled closely on the Visual Data Analysis and Reasoning (VDAR) software evaluation approach proposed by Lam et al. [41] in their overview of the possible software evaluation scenarios. The VDAR evaluation approach focused on assessing the extent to which visual analytics tools enable analysis and reasoning. The approach can be implemented using a range of methods such as ones developed to measure insight. According to Lam, VDAR studies are comparably rare in software evaluation literature and account for less than 15% of all evaluation studies published due to the challenging nature of the VDAR approach:

"Studying how a visualization tool may support analysis and reasoning is difficult since analysis processes are typically fluid and people use a large variety of approaches. In addition, the products of an analysis are difficult to standardize and quantify since both the process and its outputs are highly context-sensitive.”

Lam also noted that the few studies that do exist are often subject to issues of validity that stems, among other things, from the lack of realism in analytical task definitions, data, and workflow. Our survey of existing evaluation studies of geovisualization tools indicates that most use “locate and describe” tasks [26–28, 34]. Gomez et al., used a somewhat more complex set of look-up tasks framed on "who-when-where-what" dimensions of the data [35]. Analytical tasks that go beyond mere look-up operations and require information synthesis representative of real analytical objectives are rare; we found only two exceptions [27, 36]. The task definitions for each of these respective studies are as follows:

"Find sources, people, stories, or angles that you think could add to your coverage of the event" [following a tornado in Missouri], and

"Imagine that the State of The Union just occurred are you’re using this tool to find stories to pitch to a national news editor. Come up with at least two story angles which, with some more reporting, you think will make good news stories".

Our earlier formative evaluation of the SP3 suggests that our toolkit is best fit for the workflow requiring information foraging–finding connections among fragments of spatio-temporal information [42–44]. Using the advanced analytical tasks described above as the inspiration, we generated two realistic information foraging scenarios with two spatial data analysis tasks each for the purpose of task-based evaluation of the Co-Occurrence Matrix. Both of our scenarios position our participants in the place of an investigative journalist, whose objective is to complement their investigation of flooding events in South Carolina with social-media-based insights using the *Co-Occurrence Matrix* in SP3 and its HINM capabilities. We have used the events of the October 2015 North American storm complex (specifically, flash flooding in South Carolina in the same period) [45], with real Twitter data collected by SP3 as a dataset for this study. Our emphasis on realism (in analytical task definitions, data, and context) is a specific response to the common issues in evaluation studies reported by Lam, Bertini [41].

The actual definitions for the analytical tasks for each of the analytical scenarios are based on a unique meta-path that would provide a particular perspective into the relationships hidden in the data. The four meta-paths we have used are as follows:

*hashtag–tweet–hashtag*,
*hashtag–tweet–place mention–tweet–hashtag*,
*place mention–tweet–place mention*, *and*
*place mention–tweet–hashtag–tweet–place mention*.

The first two meta-paths treat hashtags as related if they were mentioned in the same tweet or were used to describe the same place, accordingly. The next two meta-paths treat locations as related if they were mentioned in the same tweet or were described by the same hashtag, accordingly. In order to avoid “giving away the answers”, participants in our study were introduced to the first two meta-paths as part of the tutorial but not the latter two, and the data used in the tutorial was unrelated to the data used in the actual task.

The first scenario in our study had our participants use the first and the second meta-paths to solve the following assignment:

“You are an investigative journalist. Your boss tells you he spotted a trending hashtag–#scflood–that seems to be a convenient way to collect information about the flooding events in South Carolina. He wants you to find other hashtags related to this event, so that he can more effectively track the latest developments on Twitter.”

The second scenario made use of the third and the fourth meta-paths and switched the assignment to the following:

“Your boss is happy, but he quickly finds another assignment for you. It looks like the South Carolina floods affected some key infrastructure in the state, including bridges. Your boss wants you to find a list of place mentions that come up in relationship to South Carolina floods, so that he can later investigate them in detail.”

An example of the Co-Occurrence Matrix display with the fourth meta-path selected (as seen by the study participants) is shown in Fig 5. Multiple mentions of bridges are clearly seen in this figure, a number of them associated with the “sc” and “South Carolina” columns.

Study participants were asked to document their insights, including specific hashtags and place mentions, while working through the assignments. We did not provide a specific “quota” of related entities to fill, and instead asked participants to limit themselves to 5–10 minutes per meta-path, imitating time pressure conditions of typical journalist [27]. An example of a summary generated by a participant in response to a hashtag-related query is as follows:

“#theState: Gerry Melendez, a documentary photographer from Columbia, SC noted that there appear to be high water levels in the High Congaree River, specifically under the Gervais Street bridge.”

In this example, the participant has correctly identified the hashtag “theState” as related to the South Carolina flooding events and documented specific context in which that hashtag was used. For each meta-path, we counted the total number of times each related entity was identified, with a maximum of 11 for a case when all participants successfully identified the same entity, as a way of estimating the consistency of participants’ insights.

### 3.3 Comprehension evaluation task

The last component of our user study design is a comprehension evaluation task. The purpose of the comprehension task is to provide evidence as to whether participants understand the impact of choices they make while exploring the dataset (i.e. switching from one meta-path to another), or if they simply happen to be following the instructions. We introduced this task following the advice of Gomez, Guo [35], who recommended to iterate between “controlled” and “insight” tasks while performing evaluation. The previous section described the “insight” tasks used in our study, whereas this section describes a scripted assignment with a known correct answer–a “controlled” task.

Our comprehension evaluation takes the form of an open-ended question, designed to solicit an explanation, in participants’ own terms, as to why the third and the fourth meta-paths produce drastically different results. Specifically, the question reads:

*“Based on what you’ve learned in the tutorial for this study*, *describe*, *in your own terms*, *the reason why your findings in part A of task 2* [thus, using the third meta-path] *are different from your findings in part B* [thus, using the fourth meta-path]”.

Participants were asked to document their answer to this question in writing. We have chosen meta-paths three and four because they were neither introduced nor explained as part of the tutorial, as to avoid “giving away the answers”—a common issue described by Gomez, Guo [35] and Lu, Krüger [31]. We treat the participants’ responses as correct if they contain either of the two observations:

1. Third meta-path treats locations as related if they are mentioned in the same tweet, whereas fourth meta-path treats them as related if they are share a mention of the same hashtag.
2. Third meta-path relies on tweets mentioning more than one location, which is uncommon. Fourth meta-path relies on tweets using a hashtag whenever a location is mentioned, which is more likely.

No help or guidance from the researcher was offered at this point.

The version of the study questionnaire used by the first two participants bundled the comprehension evaluation and the last analytical task together, with a single text box provided to record the answers for both tasks. Participant 2 appeared to have skipped the comprehension evaluation task, possibly due to this design choice (participant 1 was not affected). The questionnaire used by the third and subsequent participants was modified to include a separate input field for the comprehensive evaluation task.

At the end of the study session, participants were asked to fill out the remaining segments of the questionnaire corresponding to the analytical and comprehension components, consisting of a NASA TLI questionnaire and a short-answer section describing thoughts and comments (if any) about the software, the analytical tasks, and the overall study experience.

## 4. Study results

Our user study consists of three large components:

1. A hands-on, interactive tutorial,
2. Four spatial data analysis tasks, and
3. A comprehension evaluation task.

In this section, we generate a digest of the participants’ feedback corresponding to each of these components, accompanied by salient quotations, comment on consistency and correctness of participants’ performance in analytical tasks, and iterative coding of free-form study feedback.

Five of our participants were students from within the Department of Geography (none had previous exposure to the HINM-based analytical techniques or their implementation in the SP3 toolkit). We did not observe any difference in the study results between the geography and non-geography population, but we do explicitly mark the feedback from the geography students reported below using the dagger sign (†) to provide extra context for feedback interpretation.

### 4.1 Interactive tutorial feedback and results

All of the participants were able to complete the tutorial promptly (under the allotted 15 minutes) and without any issues. Written feedback can be roughly partitioned into three themes–comments on the tutorial in general, comments on the visual aids, and suggestions for improvement.

Comments on the tutorial in general were overwhelmingly positive and focused on building understanding of the function of the tool, its inner workings, and progression from simple to more advanced topics. Some of the specific remarks are as follows:

“The tutorial section was very helpful in understanding what is going on behind the matrix. […] The progression of the concepts was also executed very well”,

“The tutorial section clearly explained the logic underlying the matrix”,

“It was a good way to see how the CoMatrix is made, especially for someone who doesn’t know a ton about these things”,

“It provides a good overview for user to gain an understanding of how the tool works at a basic level” †, and

“The overall structure is reasonable, starting with easy tasks and then goes to more complicated ones.” †

A number of comments made a special point to mention the visual aids as part of the tutorial success, including remarks such as

“I really liked the visuals that went along with it, as well as the opportunity to ‘practice’”,

“It was simple, easy, and intuitive especially with diagrams” †, and

“The instructions are very clear, and the diagrams are easy to understand.” †

Surprisingly, three participants found the tutorial to be “too helpful” and expressed preference for a shorter introduction, using the following remarks:

“Although some of the actual tasks themselves seemed a little bit obvious or simple, I think it was a great instructional tool”,

“Pretty basic common sense but its good to go over to make sure everyone in the study has the same baseline knowledge on the topic”, and

“I think there was one more drawing at each stage than I needed to ‘get it,’ the last one felt repetitive.” †

The single most insightful comment for the tutorial section was as follows:

“The only concern of suggestion I would make would be in regards to clarifying a CoMatrix itself. For example, it is not very clear when a box is shaded whether the shaded box represents a) one specific tweet, or b) a relationship that exists between two or more tweets. Confusion can arise in terms of how many ‘degrees of separation’ exist between the places or hashtags listed in the CoMatrix that interest in a colored box. Otherwise, great job at explaining in a very clear manner.”

As the participant correctly guessed, the cells in the CoMatrix tool are colored according to the strength of the relationship between concepts (therefore discriminating between a single tweet and multiple tweets). Moreover, the CoMatrix (being an incomplete visual representation of a network) does not show the information about the “degrees of separation” between the concepts (i.e. the length of the meta-path used).

### 4.2 Spatial data analysis tasks feedback and results

For each meta-path, we have analyzed the participants’ findings, obtained in the form of self-reported written summaries of the related entities discovered by the participants, and counted the total number of times each related entity was identified, with a maximum of 11 for a case when all participants successfully identified the same entity. Our results indicate that participants’ findings were fairly consistent, with every entity identified by at least half of the participants, with consistency approaching 100% for a number of hashtags and place mentions, as shown in Table 1.

**Table 1:** A summary of related hashtags (top group) and related place mentions (bottom group) identified with each particular meta-path.

**#–tw–#**	**#–tw–place–tw–#**
11 theState 11 MoncksCorner 9 SCflooding 9 flood 7 congareeRiver 7 columbiasc 7 chsnews 6 Orangeburg 6 joaquin 6 Bamb(e/u)rg	11 FirstAlertWIS10 9 sctweets 7 WLTX19 6 WLTXtraffic 5 SCWX 5 SC 5 ColumbiaFlood 5 Charlestonflooding
**place–tw–place**	**place–tw–#–tw–place**
11 Columbia 11 Gervais Street Bridge	10 Wadboo Bridge 10 Congaree River 9 Cannon Bridge 9 Black River 9 Bacon Bridge 8 Saluda River 8 Limehouse Bridge 8 Eastover 8 Charleston 8 Cayce 8 Browns Ferry Bridge 7 SC 6 Georgetown

Specific meta-paths are provided as headers in bold. Corresponding counts (with the maximum of 11 possible) are listed for each of the entities, indicating the number of participants who included said entity in their task report.

### 4.3 Comprehension evaluation task results

Out of 11 participants, only two provided an incorrect answer to the question posed in this task. We treat the participants’ responses as correct if they contain either of the two observations:

1. Third meta-path treats locations as related if they are mentioned in the same tweet, whereas fourth meta-path treats them as related if they are share a mention of the same hashtag.
2. Third meta-path relies on tweets mentioning more than one location, which is uncommon. Fourth meta-path relies on tweets using a hashtag whenever a location is mentioned, which is more likely.

Correct observations falling into the first category include:

“I speculate that the findings from Part A [third meta-path] were different from Part B [fourth meta-path] in Task 2 because the first Co-Occurrence Matrix was only looking at tweets that mentioned both locations. Part B, however, was searching for related locations through the use of a hashtag”,

“Part A only looks at one tweet that mentions two locations and relates them together that way, while Part B relates tweets that have shared the same hashtag and then relates the locations, providing a lot more details and matches than Part A”, and

“The results in part A only includes tweets mentioned two locations at the same time. While part B also include locations mentioned in different tweets which are connected by the same hashtag.” †

Examples of the observations falling into the second category include:

“This would create different results in the query because individual tweets do not always use more than one location tag. Using the hashtag as the basis for searching for related places is much more fruitful in its results”,

“While Part A looks at tweets mentioning multiple places, it is not very common in tweets. So, it results in fewer co-occurrences of different places. In Part B, tweets are linked by the same hashtags, which allows inferring more associations among places” †,

“Part B generated more hits because it made hashtags the center of its search. People use hashtags when they tweet, so a search centered on hashtags is going to gather better results than a search that is centered on the contents of the tweets”, and

“[…] many tweets can have the same hashtag, but a tweet can only have so many hashtags or locations.” †

One participant made a correct guess that the second query is wider in nature due to the inclusion of a hashtags, but did not explicitly mention either of the two observations we describe above, and is therefore classified as an incorrect answer:

“By adding the elements of tweets to Part B and making the connections wider, the possibility for more blocks in CoMatrix gets bigger. The two results from Part A still came up in Part B.”

Finally, participant 2 appeared to have skipped the comprehension evaluation task. The version of the study questionnaire used by the first two participants bundled the comprehension evaluation and the last analytical task together, with a single text box provided to record the answers for both tasks, which might have caused an accidental omission on behalf of participant 2 (response by participant 1 was not affected). The questionnaire used by the third and subsequent participants was modified to include a separate input field for the comprehensive evaluation task.

### 4.4 NASA task load index (TLI) questionnaire results

**Fig 7:**
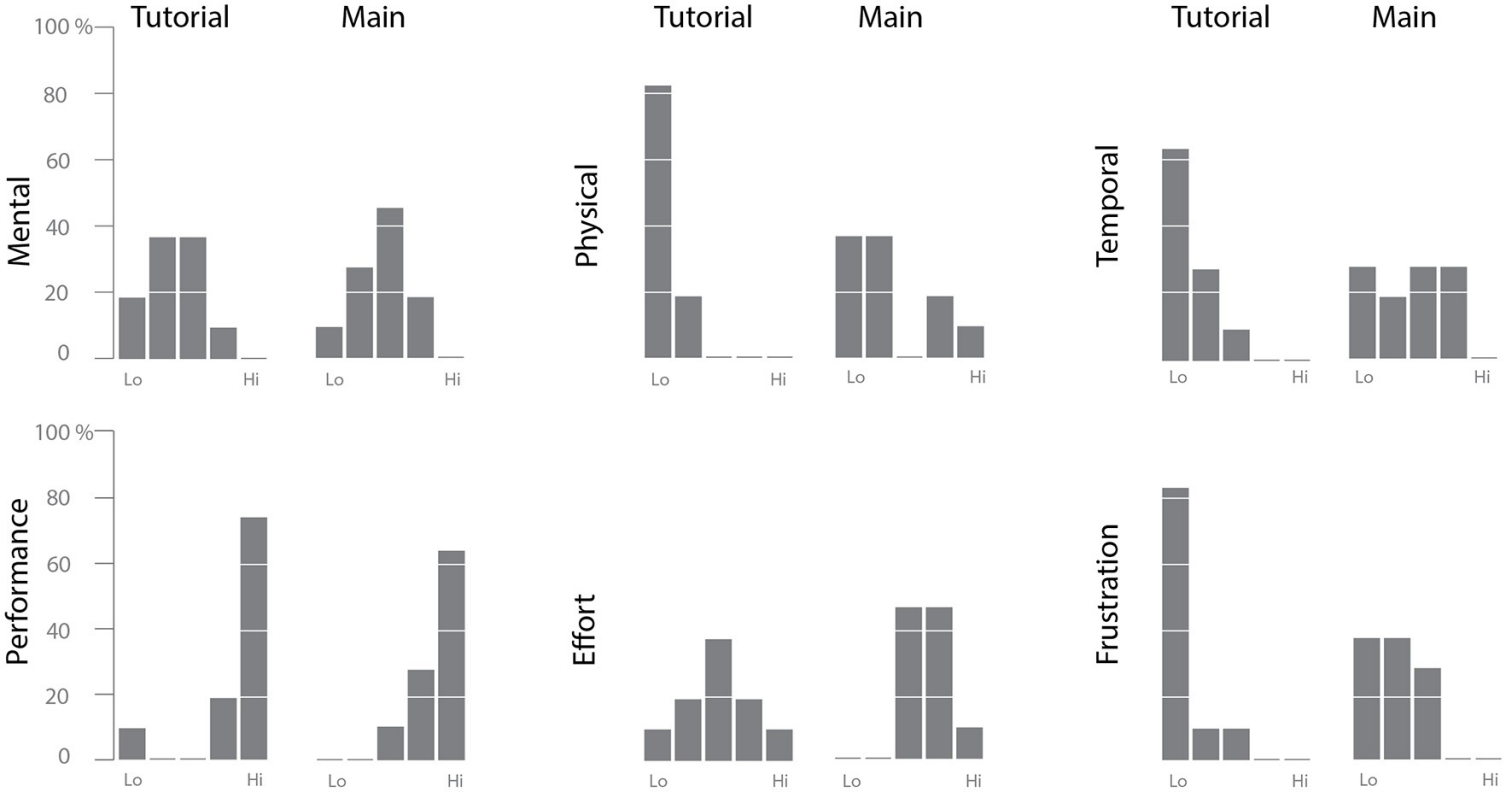
NASA Task Load Index questionnaire results. Results for both the tutorial and the main component of the user study shown for each of the 6 subscales of the Index. A low rating is good on the mental demand, physical demand, temporal demand, frustration and effort subscales (indicating low perceived workload) and a high rating is good on the performance subscale (indicating high perceived performance).

We asked participants to fill out a copy of the NASA TLI questionnaire for both the tutorial and the main component of the study. The results for both components are shown in Fig 7.

Looking at the feedback for the tutorial task, it’s clear that self-assessed level of performance was high (which is good; the one participant with a “low” performance mark had all other responses set to “low” as well, making the impression that the participant simply didn’t notice that the performance scale is inverted compared to others). Physical and temporal demand, as well as the level of frustration, were low across the board (which is good), and mental demand and total effort have a somewhat normal distribution of scores.

Looking at the change in TLI scores from the tutorial to the main part of the study, there is a slight shift towards higher perceived workload across the board. Most notable is the shift in physical and temporal demand, which likely corresponds to the fact that participants were given a self-imposed time limit for each task and had to actively interact with the system, compared to the relaxed pace and minimal amount of physical activity (drawing) required by the tutorial. Self-assessed level of performance remains very high, although for most participants that came at the expense of at least moderate effort and at least some (although still low) frustration.

### 4.5 Overall study feedback

At the conclusion of the study, participants were asked to provide any remaining (free-form) feedback about their experience. This section presents a digested summary of those comments, starting with feedback about the study design as a whole.

We mentioned in Section 3.1 that tutorial diagrams were purposefully oriented in a way that placed hashtags along the margins of the Co-Occurrence Matrix to make the connection between the network-like diagrams and the CoMatrix explicit. One of the participants recognized this strategy and left a comment praising this specific decision:

“Its good that the tool is set up how you would make the diagram, otherwise that would get confusing”.

Another insightful comment was made about the meta-path selection tool (seen on top of Fig 5), which is implemented as a series of drop-down menus that are used to build a specific meta-path step by step:

“When you describe using the [meta-]path tool in the instructions, maybe provide more thorough instructions. I was confused when it appeared it wanted me to select an answer with multiple options, without knowing that once I clicked the first one, I would be able to select more after.”

Perhaps even more importantly, another participants remarked that

“I appreciated having a variety of ‘paths’ available to explore a certain topic. My only concern would be if I was asked to do this myself, I might not know exactly what paths to investigate in order to be sure to not miss any information”.

Both of these comments highlight the fact that this study was primarily aimed at evaluating the plausibility (cognitive and analytical) of integrating heterogeneous network mining as part of the geovisual analytics framework. A larger problem, requiring much further investigation, is the identification of optimal visual metaphors that could be used to represent meta-paths, their relationship to available data, and the various properties of the connections that exist within the spatial datasets under investigation.

Aside from the comments on their overall experience, some participants provided insightful feedback about the design of our software, which might be of relevance to those building comparable systems. For example, although most participants praised the system for ease of use, we got conflicting feedback regarding the coordinated view approach with multiple windows (showing the CoMatrix in a browser pop-up while the rest of the SensePlace3 UI is shown in a separate window). One participant remarked that

“the co-occurrence matrix was very easy to use, and I was able to line the tweet window up just to the side of the matrix so that I could see all of the windows in my workspace”,

whereas another participant suggested that

“it would be better if the co-matrix window could itself be integrated into the main senseplace application. This would negate the need to switch back and forth between windows and may improve user performance” †.

During the experiment, we have provided ample screen space to ensure that all of the application windows fit at once, so the choice between single- and multiple-window workflow might be subject to personal preferences.

A number of participants expressed complaints about small font size for the Co-Occurrence Matrix component, making remarks such as

“The only thing that was a little difficult is the small text in the matrix. Sometimes the letters would be smooshed together”,

“The placement of the words on the top is hard to recognize, especially considering some of hashtags are meaningless. So sometimes I have to click on the tweet to figure out what are those words” †, or

“The font at the top was also very hard to read, you might want to test something else that’s more readable tilted.” †

Although this is a valid usability concern, it is also the most trivial to fix–SP3 supports the full set of browser functions, including making the font larger through browser controls.

Another usability issue brought up by some participants refers to the CoMatrix arranging rows and columns according to data properties (e.g. frequency of hashtag mention), but not necessarily according to current user needs:

“For the CoMatrix, it would be better that I can manually change a little bit about the ordering” †, or

“It was difficult to keep track of South Carolina, which was deep in the matrix–the rows blend together. I would have wider grid lines every 6 rows, or some other visual element to help the eye track of locations in the grid.” †

This is, of course, an idea that has been used in many past systems and traces at least to Bertin’s Semiology of Graphics [17] in which he discusses matrix manipulation and a manually sortable matrix tool designed in his laboratory. Adding both manual and computational row-column sorting to the CoMatrix is a task for future development.

Two participants made a comment about what are apparently a set of data issues. One participant remarked that

“Many of the tweets shown in the application were no longer up or accessible [when a link to the original message on Twitter is clicked]” †.

This issue will be unavoidable for any system using real Twitter data, and, despite having relevant tweets in our archive, we cannot guarantee that the original owner still maintains their Twitter account with every tweet intact. Another participant reported that

“[CoMatrix generated] some false positive when […] using a more general relation.” †

This issue highlights the messy nature of real data, with unrelated hashtags and place mentions surfacing in response to flood-related queries. Although this issue is both significant and challenging, it does not invalidate the HINM-based geovisualization approach as a strategy for sifting through messy real-world data and achieving insights in spite of the irrelevant data.

## 5. Discussion

This study demonstrates that an analyst with minimal (15 minutes or less) training can comprehend the HINM metaphors (as implemented in SensePlace3), can employ said metaphors to explore real spatial data, and can successfully interpret the results of such spatial analyses. Beyond this core contribution, we highlight a number of other observations below that are either suggested by or can be directly derived from the study results.

### 5.1 The role of the advanced tutorial component

In an earlier formative evaluation of the SP3, we have obtained a range of broad feedback concerning the usability of each of its individual components [1]. CoMatrix was ranked as the least usable component at the time, with 44% of participants describing it as “difficult” to use, and 22% as “very difficult”. Although participants agreed that the CoMatrix component has potential for information foraging tasks, many pointed out that specialized training might be necessary, including instruction on “how to interpret what it contains”. By comparison, the results reported in Section 4.3 (Comprehension evaluation task) demonstrate that nine of the eleven participants were able to make logical inferences using HINM concepts without external assistance, and Section 4.4 (NASA TLI scores) documents high level of self-assessed performance and low level of frustration during all study tasks. Finally, the results reported in Sections 4.1 (Tutorial feedback) of this study indicate that participants found our tutorial component both instructive and even enjoyable, with one of the participants having deduced (on her own and without an external prompt) that CoMatrix is an incomplete visual representation of a network–an impressive conclusion as we purposefully omit these advanced concepts to simplify the tutorial material. Combined, we interpret the above as tentative evidence for the importance of a hands-on tutorial and a graphical approach to explaining data modeling metaphors in the successful adoption of advanced data mining techniques. An important caveat is that the current study does not contain a control group (e.g. one without hands-on tutorial or a graphical approach), and the earlier formative study was neither conceptualized nor designed to form a cohesive series with the current investigation, hence additional work is needed to draw a robust conclusion.

### 5.2 HINM approach in (geo)visual analytics—A case study

Although this study does not aim to validate HINM as a data modeling approach (there is considerable evidence for this in the existing literature, as cited above), the spatial data analysis tasks described in Section 3.2 were designed in such way that specific findings obtained by participants would form a miniature *case study*. This case study would then generate tentative evidence for the applicability and utility of the HINM-based approach (made accessible through a visual interface that provides immediate visual feedback for user queries) to the problems of spatial data analysis.

The results reported in Section 4.2 demonstrate that our participants were able to use the HINM metaphors to explore real (messy and ambiguous) geospatial data, with a certain level of consistency evident across the documented insights. It is worth noting that a number of place mentions reported in Table 1 in Section 4.2 were correctly identified as affected by the South Carolina floods despite the fact that their location is potentially ambiguous, including areas such as *Cannon Bridge*, *Charleston* and *Cayce*, which are all valid places in at least one other state besides South Carolina. This would not be possible in the traditional SP3 workflow as it usually relies on GeoTxt, a state of the art tool for automatic geocoding of place mentions [14], for spatial analysis, and such automatic tools do not cope well with data ambiguity. For example, top picks for *Georgetown* and *Eastover* in the GeoNames gazetteer (another prominent database used for geocoding, see http://www.geonames.org/) are located in Texas and North Carolina, respectively. GeoNames also lists *Black River* as a populated place in New York and Wisconsin, a stream in Michigan, Arkansas and Minnesota, and a township in North Carolina prior to describing it as a stream in South Carolina.

We were surprised to discover that the Co-Occurrence Matrix tool was able to correctly link all of these places to the South Carolina flooding events. In retrospect, this is likely due to the inclusion of the hashtags as the central link in one of the meta-paths we have used (*place mention–tweet–hashtag–tweet–place mention*). A flood-related hashtag, used to describe both unambiguous place mentions (e.g. *South Carolina*) as well as the ambiguous ones (e.g. *Black River*) would then be used to establish a clear link between the two, eliminating the spatial ambiguity of the original reference.

Combined, we interpret the above as tentative evidence that the HINM approach might produce a positive difference in the outcome of spatial analysis involving messy data with complex interconnections (when made accessible through a visual interface that provides immediate visual feedback for user queries), and has additional potential for geoparsing and geolocation applications, addressing the challenges identified in related work [46, 47].

### 5.3 Minor contributions

The second half of Section 4.5 outlines a number of comments pertaining to the design of our software, which might be of relevance to those building comparable systems. Having participants remark on minor items such as font size or placement of the CoMatrix on the screen (in a separate window or embedded with the rest of the SP3 interface) might be seen in a positive light, indicating that the system functioned as expected with no major breakdowns in its analytical functionality. On the other hand, minor incremental improvement might still be in order as one of the conditions of a successful VDAR study is that the overall system experience had reached the level where participants can focus on the analytical tasks without any distractions, software-induced or otherwise [41]–a high bar to clear.

In Section 4.4, we report the results of our assessment of the workload associated with the use of advanced HINM tools in the (geo)visual analytics workflow. This assessment addresses prior contentions that social media data require a workload that may make their use impractical for real-world adoption in time-critical situations. Our results demonstrate that the workload demands of using SP3 for HINM-based analysis are judged to be relatively low and that even for complex analytical tasks the HINM-based visual interface can generate high level of self-perceived performance, accompanied by a low level of frustration–a positive sign.

While not investigated directly in this research, we hypothesize that tools that represent and expose the complex connections that permeate heterogeneous linked data and that allow users to forage across those connections can support associative systems of reasoning. Sloman [48] contends that "Associative systems can capitalize on the ability of memory and similarity-based generalization to usually draw reasonable inferences, while maintaining the flexibility to do so in uncountable varieties of situations." Thus, associative reasoning is the process that is critical to effective sensemaking with messy, heterogeneous data. Ideas presented here on enabling heterogeneous information network mining are also compatible with a "cognitive network model of creativity" proposed by Santanen, et al [49]. Thus, the potential of the approach to support creativity through exposing unanticipated connections across information nuggets is a potentially interesting avenue for future research.

Finally, we make a methodological contribution to the field of (geo)visual analytics by means of generating and documenting a user study protocol that is based on and improves upon the current methodological state of the art. Detailed evaluation protocols are rare in (geo)visual analytics research, making the proposed protocol a tangible contribution to our field of study.

### 5.4 Study limitations

Current implementation of the web-based HINM system is a prototype built to explore the potential and challenges associated with integration of the heterogeneous network modelling approach into interactive, web-based visual analytical environments that focus on geographic data. Despite its strengths, this prototype is still limited in a number of ways.

One such limitation was previously highlighted by a participant as part of the study feedback in Section 4.1. Specifically, the Co-Occurrence Matrix does not currently communicate neither the length nor the “shape” of connections that are presented in aggregate terms by means of adjusting the color (or other attributes) of the CoMatrix cells. Although our experiment was a success, it is entirely plausible that adding these extra dimensions to the display might further enhance the capacity of the analyst to investigate the phenomena at hand, hence additional conceptual and software development work is needed in order to identify the optimal visual metaphors that could be used to represent meta-paths, their relationship to available data, and the various properties of the connections that exist within the spatial datasets under investigation. Additionally, computational methods should be investigated to suggest meta-paths having a high probability of interesting and useful results–a concern also reflected in user feedback in Section 4.5. Such computational method could build upon ideas from projection pursuit in statistics [50] and recommender systems used in many big data query situations [51].

Another limitations is the size of the dataset explored in this study. We currently only use the SensePlace3 query results (limited to about 1,000 tweets) to build the heterogeneous networks used throughout the evaluation process. Building a complete network from our entire multi-year data archive will require significant investments into graph database technology and accompanying hardware, which was deemed unwise before the potential utility of the resulting analytical system has been fully evaluated. This, however, is not a significant limitation, as HINM has been previously verified as a computationally viable technique when applied to much larger datasets. Furthermore, recent success stories in modelling of large-scale networks on commodity hardware [52] make it clear that this approach is fully compatible with challenges posed by Big Data.

## Supporting information

S1 File Facsimile of the study hand-out. This file contains the facsimile of the study tutorial and analytical task definitions.
(DOCX)

S2 File Facsimile of the feedback questionnaire. (DOCX)

S3 File Complete questionnaire results. This file contains, in a compressed format, the raw data provided by the participants of the study by means of the study questionnaire.
(ZIP)
