# Supplementary material for: Augmenting geovisual analytics of social media data with heterogeneous information network mining—Cognitive plausibility assessment
Source: PLoS One. 2018 Dec 4;13(12):e0206906. doi: 10.1371/journal.pone.0206906 (PMC6279051; doi:10.1371/journal.pone.0206906)
Supplement: S1 File — This file contains the facsimile of the study tutorial and analytical task definitions. (DOCX) [file pone.0206906.s001.docx]

# Advanced Co-Matrix Tutorial

# Part A

#hashtag2

#hashtag1

tweet

1. Convert the following three tweets into hashtag diagrams:
   1. "Heavy rain in Westmoreland cnty #paWeather #rain"
   2. "Heavy rain continues, chances of flooding in Westmoreland cnty #paWeather #flood"
   3. "Multiple Westmoreland bridges in danger of flooding #bridge #flood"
2. Using the diagrams above, fill in the cells in the miniature co-matrix visualization below:

|  | **#rain** | **#flood** |
| --- | --- | --- |
| **#paWeather** |  |  |
| **#bridge** |  |  |

# Part B

1. Convert the following four tweets into hashtag diagrams:
   1. "Avoid Westmoreland co., more rain later in the day #rain"
   2. "Westmoreland co. issues official flooding warning #flood"
   3. "Part of I-76 flooded, just saw a huge pile-up happen #accident"
   4. "Westmoreland co. announces blood drive for those injured in I-76 hwy pile-up #bloodDrive"

**a-b:**

**a-c:**

**a-d:**

**b-c:**

**b-d:**

**c-d:**

1. Using the diagrams above, fill in the cells in the miniature co-matrix visualization below:

|  | **#flood** | **#bloodDrive** |
| --- | --- | --- |
| **#rain** |  |  |
| **#flood** |  |  |
| **#accident** |  |  |

# Tutorial summary

In the parts A and B of this tutorial, you built two co-matrix visualizations.

The first co-matrix shows relationships between hashtags used in the same tweet (with your diagrams taking shape of “hashtag – tweet – hashtag”).

The second co-matrix shows relationships between hashtags in tweets *that mention the same place* (with your diagrams taking shape of “hashtag – tweet – place mention – tweet – hashtag”).

The same procedure can be used to build co-matrices that show relationships between place mentions (rather than between hashtags), as shown below.

place mention 2

place mention 1

tweet

#hashtag2

#hashtag1

tweet

shared location

#hashtag2

tweet

#hashtag1

tweet

shared #hashtag

place mention 2

tweet

place mention 1

tweet

Provide feedback about this tutorial before proceeding to the next task.

# Task 1 – Hashtags and Floods

For this and every other task, start by reading the entire page. Once you read everything, return to the first item in the list below and proceed to carry out the task. You are welcome to ask questions at any point.

You are an investigative journalist. Your boss tells you he spotted a trending hashtag – #scflood – that seems to be a convenient way to collect information about the flooding events in South Carolina. He wants you to find other hashtags related to this event, so that he can more effectively track the latest developments on Twitter.

# Part A

1. Locate the “network path” tool at the top of the co-matrix window.
2. Using the path tool, select “hashtags – tweets – hashtags” as the way to build the co-matrix visualization.
3. Sort the co-matrix by frequency of mention.
4. Find the #scflood hashtag in the left co-matrix column (it should be at the top).
5. Scanning along the #scflood row, identify other hashtags related to the South Carolina flood (click on the individual cells to investigate ambiguous hashtags).
6. Using the study questionnaire, write down the hashtags you found along with a brief (a sentence or less) description of their meaning, where possible.

# Part B

1. In the same co-matrix display, click "reset path" next to the path tool.
2. Using the path tool, select “hashtags – tweets – about locations – tweets – hashtags” as the way to build the co-matrix visualization.
3. Find the #scflood hashtag in the left co-matrix column.
4. Scanning along the #scflood row, identify other hashtags related to the South Carolina flood (click on the individual cells to investigate ambiguous hashtags).
5. Using the study questionnaire, write down the hashtags you found, **excluding the hashtags you identified in the part A of this task**. Where possible, include a brief (a sentence or less) description of the hashtag meaning.

# Task 2 – South Carolina Bridges

Your boss is happy, but he quickly finds another assignment for you. It looks like the South Carolina floods affected some key infrastructure in the state, including bridges. Your boss wants you to find a list of place mentions that come up in relationship to South Carolina floods, so that he can later investigate them in detail.

# Part A

1. In the same co-matrix display, click "reset path" next to the path tool.
2. Using path tool, select “about locations – tweets – about locations” as the way to build the co-matrix visualization.
3. Sort the matrix by name.
4. Find the words “South Carolina” in the left co-matrix column.
5. Scanning along the South Carolina row, identify related place mentions (click on the cells to investigate ambiguous places).
6. Using the study questionnaire, write down the place mentions you found. Include a brief (a sentence or less) description of what those places are, where possible.

# Part B

1. In the same co-matrix display, click "reset path" next to the path tool.
2. Using the path tool, select “about locations – tweets – hashtags – tweets – about locations” as the way to build the co-matrix visualization.
3. Find the words “South Carolina” in the left column.
4. Scanning along the South Carolina row, identify related place mentions (click on the cells to investigate ambiguous places).
5. Using the study questionnaire, write down the place mentions you found, **excluding the place mentions you identified in the part A of this task**. Where possible, include a brief (a sentence or less) description of what those places are.

# Part C

1. Based on what you've learned in the tutorial for this study, describe, in your own terms, the reason why your findings in part A of task 2 are different from your findings in part B. You are welcome to revisit the tutorial materials while doing so.
2. Provide feedback about tasks 1 and 2 using the questionnaire.
