# Supplementary material for: Augmenting geovisual analytics of social media data with heterogeneous information network mining—Cognitive plausibility assessment
Source: PLoS One. 2018 Dec 4;13(12):e0206906. doi: 10.1371/journal.pone.0206906 (PMC6279051; doi:10.1371/journal.pone.0206906)
Supplement: S3 File — This file contains, in a compressed format, the raw data provided by the participants of the study by means of the study questionnaire. (ZIP) [file pone.0206906.s003.zip › questionnaireResults/questionnaire.netw.1.docx]

# Tutorial Feedback

Describe the level of mental demand for the tutorial tasks (e.g. amount of thinking, remembering, searching, etc.):

| Low |  |  |  | High |
| --- | --- | --- | --- | --- |
|  |  |  |  |  |

Describe the level of physical demand for the tutorial tasks (e.g. amount of clicking, scrolling, typing, etc.):

| Low |  |  |  | High |
| --- | --- | --- | --- | --- |
|  |  |  |  |  |

Describe the level of temporal demand for the tutorial tasks (i.e. the amount of time pressure you experienced):

| Low |  |  |  | High |
| --- | --- | --- | --- | --- |
|  |  |  |  |  |

Describe your level of performance for the tutorial tasks (i.e. how much success you think you had in accomplishing the goals of this task):

| Low |  |  |  | High |
| --- | --- | --- | --- | --- |
|  |  |  |  |  |

Describe the amount of effort you put into the tutorial tasks to achieve your level of performance:

| Low |  |  |  | High |
| --- | --- | --- | --- | --- |
|  |  |  |  |  |

Describe the amount of frustration you experienced during the tutorial tasks:

| Low |  |  |  | High |
| --- | --- | --- | --- | --- |
|  |  |  |  |  |

Please describe thoughts and comments (if any) that you have about the tutorial section (related to individual tasks, overall structure, etc.):

| None. |
| --- |

# Task 1 – Hashtags and Floods

Please enter your findings from **Part A** of this task in the box below:

| Some of the hashtags that I found were #chstrfc, #chsnews, #hrtraffic, and #flooding. The meanings of these hashtags reveal a specific location of the SC flood, which is mainly Charleston. It also reveals that there are traffic problems that are being caused by the flood. |
| --- |

Please enter your findings from **Part B** of this task in the box below:

| Some of the hashtags that I found were #FirstAlertWIS10, #congaeriver, and #chsnews. The meanings of these hashtags show that there are news alert stations that are reporting the status of the flood. In addition, the mention of the Congae River could potentially hint that the flood has caused massive problems to areas near it. |
| --- |

# Task 2 – South Carolina Bridges

Please enter your findings from **Part A** of this task in the box below:

| The places that I found from the hashtags were Columbia and Gervais Street Bridge. The mention of Columbia seems to be a town or city in South Carolina that was affect heavily by the flood. The mention of Gervais Street Bridge could potentially mean that there are damages to the bridge due to the flood and as a result, it is closed down for repairs. |
| --- |

Please enter your findings from **Part B** of this task in the box below:

| The places that I found from the hashtags were Bacon Bridge, Black River, Browns Ferty Bridge and Cannon Bridge. These locations reveal places in South Carolina that were affected by the flood. Perhaps it means that these bridges or rivers have suffered severe damages and have caused problems for inhabitants living near these areas.  The reason that I think the findings from in Part A of task 2 are different from findings in Part B is that Part A looks for tweets with two hashtags to mention different locations and then creates a relationship between the two hashtags. However, part B looks for two tweets who are connected by an identical word or phrase but uses two different hashtags. Part B then creates a relationship between the two different hashtags to refer to one similar event, which in this case was the South Carolina flood. |
| --- |

# Joint Feedback for Tasks 1 and 2

Describe the level of mental demand for these tasks (e.g. amount of thinking, remembering, searching, etc.):

| Low |  |  |  | High |
| --- | --- | --- | --- | --- |
|  |  |  |  |  |

Describe the level of physical demand for these tasks (e.g. amount of clicking, scrolling, typing, etc.):

| Low |  |  |  | High |
| --- | --- | --- | --- | --- |
|  |  |  |  |  |

Describe the level of temporal demand for these tasks (i.e. the amount of time pressure you experienced):

| Low |  |  |  | High |
| --- | --- | --- | --- | --- |
|  |  |  |  |  |

Describe your level of performance for these tasks (i.e. how much success you think you had in accomplishing the goals of this task):

| Low |  |  |  | High |
| --- | --- | --- | --- | --- |
|  |  |  |  |  |

Describe the amount of effort you put into these tasks to achieve your level of performance:

| Low |  |  |  | High |
| --- | --- | --- | --- | --- |
|  |  |  |  |  |

Describe the amount of frustration you experienced during these tasks:

| Low |  |  |  | High |
| --- | --- | --- | --- | --- |
|  |  |  |  |  |

Describe specific ways, if any, in which individual tool features helped or hampered your progress in these tasks:

| None. |
| --- |

Please describe any additional thoughts that were not covered by the previous questions (including thoughts about SensePlace3, individual tasks, the study as a whole, etc.):

| None. |
| --- |

You are done! Check in with the scientist to receive your payment.
