# Supplementary material for: Augmenting geovisual analytics of social media data with heterogeneous information network mining—Cognitive plausibility assessment
Source: PLoS One. 2018 Dec 4;13(12):e0206906. doi: 10.1371/journal.pone.0206906 (PMC6279051; doi:10.1371/journal.pone.0206906)
Supplement: S3 File — This file contains, in a compressed format, the raw data provided by the participants of the study by means of the study questionnaire. (ZIP) [file pone.0206906.s003.zip › questionnaireResults/questionnaire.netw.10.docx]

# Tutorial Feedback

Describe the level of mental demand for the tutorial tasks (e.g. amount of thinking, remembering, searching, etc.):

| Low |  |  |  | High |
| --- | --- | --- | --- | --- |
|  |  |  |  |  |

Describe the level of physical demand for the tutorial tasks (e.g. amount of clicking, scrolling, typing, etc.):

| Low |  |  |  | High |
| --- | --- | --- | --- | --- |
|  |  |  |  |  |

Describe the level of temporal demand for the tutorial tasks (i.e. the amount of time pressure you experienced):

| Low |  |  |  | High |
| --- | --- | --- | --- | --- |
|  |  |  |  |  |

Describe your level of performance for the tutorial tasks (i.e. how much success you think you had in accomplishing the goals of this task):

| Low |  |  |  | High |
| --- | --- | --- | --- | --- |
|  |  |  |  |  |

Describe the amount of effort you put into the tutorial tasks to achieve your level of performance:

| Low |  |  |  | High |
| --- | --- | --- | --- | --- |
|  |  |  |  |  |

Describe the amount of frustration you experienced during the tutorial tasks:

| Low |  |  |  | High |
| --- | --- | --- | --- | --- |
|  |  |  |  |  |

Please describe thoughts and comments (if any) that you have about the tutorial section (related to individual tasks, overall structure, etc.):

| I really liked the visuals that went along with it, as well as the opportunity to “practice.” It was a good way to see how the co-matrix is made, especially for someone who doesn’t know a ton about these things. |
| --- |

# Task 1 – Hashtags and Floods

Please enter your findings from **Part A** of this task in the box below:

| #chsnews- Charleston News  #thestate  #MoncksCorner  #Orangeburg- Orangeburg county  #Bamberg- Bamberg county  #flood- flood  #joaquin  #SCflooding  #columbiasc  #congaeeriver |
| --- |

Please enter your findings from **Part B** of this task in the box below:

| #FirstAlertWIS10- TV station news?  #chstrfc  #sctweets  #WLTX19- radio station?  #WLTXtraffic- traffic updates  #project365  #day274 |
| --- |

# Task 2 – South Carolina Bridges

Please enter your findings from **Part A** of this task in the box below:

| Columbia- Columbia, SC, the capitol of the state  Gervais Street Bridge- bridge in South Carolina |
| --- |

Please enter your findings from **Part B** of this task in the box below:

| Bacon Bridge- bridge in SC  Black River- river in SC  Browns Ferry Bridge- bridge in SC  Cannon Bridge- bridge in SC  Cayce- town in SC  Charleston- town in SC  Congaree- river in SC  Eastover  Georgetown- county in SC  Limehouse Bridge- bridge in SC  Saluda River- river in SC  SC- South Carolina  Wadboo Bridge- bridge in SC  West Columbia |
| --- |

Please enter your findings from **Part C** of this task in the box below:

| By adding the elements of tweets to Part B and making the connections wider, the possibility for more blocks in co-matrix gets bigger. The two results from Part A still came up in Part B. |
| --- |

# Joint Feedback for Tasks 1 and 2

Describe the level of mental demand for these tasks (e.g. amount of thinking, remembering, searching, etc.):

| Low |  |  |  | High |
| --- | --- | --- | --- | --- |
|  |  |  |  |  |

Describe the level of physical demand for these tasks (e.g. amount of clicking, scrolling, typing, etc.):

| Low |  |  |  | High |
| --- | --- | --- | --- | --- |
|  |  |  |  |  |

Describe the level of temporal demand for these tasks (i.e. the amount of time pressure you experienced):

| Low |  |  |  | High |
| --- | --- | --- | --- | --- |
|  |  |  |  |  |

Describe your level of performance for these tasks (i.e. how much success you think you had in accomplishing the goals of this task):

| Low |  |  |  | High |
| --- | --- | --- | --- | --- |
|  |  |  |  |  |

Describe the amount of effort you put into these tasks to achieve your level of performance:

| Low |  |  |  | High |
| --- | --- | --- | --- | --- |
|  |  |  |  |  |

Describe the amount of frustration you experienced during these tasks:

| Low |  |  |  | High |
| --- | --- | --- | --- | --- |
|  |  |  |  |  |

Describe specific ways, if any, in which individual tool features helped or hampered your progress in these tasks:

| The tutorials were a big help. They helped me understand how these tools were created and how the results on the co-matrix are found. The co-matrix was also good, but I found that results sometimes didn’t match with the boxes clicked. |
| --- |

Please describe any additional thoughts that were not covered by the previous questions (including thoughts about SensePlace3, individual tasks, the study as a whole, etc.):

| This tool is very cool and I think it could be of use along a number of fields. |
| --- |

You are done! Check in with the scientist to receive your payment.
