# Supplementary material for: Augmenting geovisual analytics of social media data with heterogeneous information network mining—Cognitive plausibility assessment
Source: PLoS One. 2018 Dec 4;13(12):e0206906. doi: 10.1371/journal.pone.0206906 (PMC6279051; doi:10.1371/journal.pone.0206906)
Supplement: S3 File — This file contains, in a compressed format, the raw data provided by the participants of the study by means of the study questionnaire. (ZIP) [file pone.0206906.s003.zip › questionnaireResults/questionnaire.netw.11.docx]

# Tutorial Feedback

Describe the level of mental demand for the tutorial tasks (e.g. amount of thinking, remembering, searching, etc.):

| Low |  |  |  | High |
| --- | --- | --- | --- | --- |
|  |  |  |  |  |

Describe the level of physical demand for the tutorial tasks (e.g. amount of clicking, scrolling, typing, etc.):

| Low |  |  |  | High |
| --- | --- | --- | --- | --- |
|  |  |  |  |  |

Describe the level of temporal demand for the tutorial tasks (i.e. the amount of time pressure you experienced):

| Low |  |  |  | High |
| --- | --- | --- | --- | --- |
|  |  |  |  |  |

Describe your level of performance for the tutorial tasks (i.e. how much success you think you had in accomplishing the goals of this task):

| Low |  |  |  | High |
| --- | --- | --- | --- | --- |
|  |  |  |  |  |

Describe the amount of effort you put into the tutorial tasks to achieve your level of performance:

| Low |  |  |  | High |
| --- | --- | --- | --- | --- |
|  |  |  |  |  |

Describe the amount of frustration you experienced during the tutorial tasks:

| Low |  |  |  | High |
| --- | --- | --- | --- | --- |
|  |  |  |  |  |

Please describe thoughts and comments (if any) that you have about the tutorial section (related to individual tasks, overall structure, etc.):

| I think there was one more drawing at each stage than I needed to “get it,” the last one felt repetitive. |
| --- |

# Task 1 – Hashtags and Floods

Please enter your findings from **Part A** of this task in the box below:

| SCFloods is associated with #thestate and #MoncksCorner the most, with two co-occurences with #SCflooding.  #thestate, referse to SC doesn’t seem to be a common hashtag, just part of a single tweet that has been re-tweeted several times (which warned of high water levels under the Gervais Street bridge.)  #MoncksCorner- Specific location- similar to above, occurs often because of multiple retweets. Also concerns a flooding bridge, Wadboo Bridge  #SCflooding- event, but same as above, but for Hancock Bridge  One co-occurrence also happened with the following: each with #chsnews (new source), #flood (event), #joaquin (storm), #orangeburge, #Bamburg, #columbiasc, and #cognare river (locations) |
| --- |

Please enter your findings from **Part B** of this task in the box below:

| FirstAlertWIS10- news service  Chsfric- Charleston-related, tweets correspond to Limehouse bridge closure  Sctweets- south Carolina tweets  WTLX19- news source  WTLCtraffic- news source  Less-  Project365- I think this is a phenomenon where people post a picture a day?  Day274- a single day in the above phenomenon  Jobs- THEY TOOK ‘EM  Jobsfairusa- event  Careerbuilder- web service  Scwx- South Carolina Weather  Columbiaflood- event  Charlestonflooding- event  Sc- South Carolina |
| --- |

# Task 2 – South Carolina Bridges

Please enter your findings from **Part A** of this task in the box below:

| Columbia- specific city  Gervais Street bridge- bridge damaged or threatened by flooding |
| --- |

Please enter your findings from **Part B** of this task in the box below:

| Bacon Bridge- bridge  Black River- river  Brows Ferry Bridge- bridge  Cannon Bridge- bridge  Cayce- City/Suburb?  Charleston- City  Columbia- City  Congaree- River  Easover- City  Georgetown- County  Limehouse Bridge- bridge  Saluda River- river  SC- State  Wadboo Bridge- bridge  West Columbia- city/suburb |
| --- |

Please enter your findings from **Part C** of this task in the box below:

| It’s a larger network since it’s based on many hashtags (many-to-many), rather than tweets (one-to-many, and not even *that* many).  In other words, many tweets can have the same hashtag, but a tweet can only have so many hashtags or locations. This means that the dataset is much larger in Part B. |
| --- |

# Joint Feedback for Tasks 1 and 2

Describe the level of mental demand for these tasks (e.g. amount of thinking, remembering, searching, etc.):

| Low |  |  |  | High |
| --- | --- | --- | --- | --- |
|  |  |  |  |  |

Describe the level of physical demand for these tasks (e.g. amount of clicking, scrolling, typing, etc.):

| Low |  |  |  | High |
| --- | --- | --- | --- | --- |
|  |  |  |  |  |

Describe the level of temporal demand for these tasks (i.e. the amount of time pressure you experienced):

| Low |  |  |  | High |
| --- | --- | --- | --- | --- |
|  |  |  |  |  |

Describe your level of performance for these tasks (i.e. how much success you think you had in accomplishing the goals of this task):

| Low |  |  |  | High |
| --- | --- | --- | --- | --- |
|  |  |  |  |  |

Describe the amount of effort you put into these tasks to achieve your level of performance:

| Low |  |  |  | High |
| --- | --- | --- | --- | --- |
|  |  |  |  |  |

Describe the amount of frustration you experienced during these tasks:

| Low |  |  |  | High |
| --- | --- | --- | --- | --- |
|  |  |  |  |  |

Describe specific ways, if any, in which individual tool features helped or hampered your progress in these tasks:

| It was difficult to keep track of South Carolina, which was deep in the matrix- the rows blend together. I would have wider grid lines every 6 rows, or some other visual element to help the eye track of locations in the grid. The font at the top was also very hard to read, you might want to test something else that’s more readable tilted, or adjust the kerning. I had to re-type a lot of my hashtags after I zoomed in.  Linked screen is nice.  Colors are nice, but some of the categories are close in brightness- well, are they classed or continuous? |
| --- |

Please describe any additional thoughts that were not covered by the previous questions (including thoughts about SensePlace3, individual tasks, the study as a whole, etc.):

|  |
| --- |

You are done! Check in with the scientist to receive your payment.
