# Supplementary material for: Augmenting geovisual analytics of social media data with heterogeneous information network mining—Cognitive plausibility assessment
Source: PLoS One. 2018 Dec 4;13(12):e0206906. doi: 10.1371/journal.pone.0206906 (PMC6279051; doi:10.1371/journal.pone.0206906)
Supplement: S3 File — This file contains, in a compressed format, the raw data provided by the participants of the study by means of the study questionnaire. (ZIP) [file pone.0206906.s003.zip › questionnaireResults/questionnaire.netw.2.docx]

# Tutorial Feedback

Describe the level of mental demand for the tutorial tasks (e.g. amount of thinking, remembering, searching, etc.):

| Low |  |  |  | High |
| --- | --- | --- | --- | --- |
|  |  |  |  |  |

Describe the level of physical demand for the tutorial tasks (e.g. amount of clicking, scrolling, typing, etc.):

| Low |  |  |  | High |
| --- | --- | --- | --- | --- |
|  |  |  |  |  |

Describe the level of temporal demand for the tutorial tasks (i.e. the amount of time pressure you experienced):

| Low |  |  |  | High |
| --- | --- | --- | --- | --- |
|  |  |  |  |  |

Describe your level of performance for the tutorial tasks (i.e. how much success you think you had in accomplishing the goals of this task):

| Low |  |  |  | High |
| --- | --- | --- | --- | --- |
|  |  |  |  |  |

Describe the amount of effort you put into the tutorial tasks to achieve your level of performance:

| Low |  |  |  | High |
| --- | --- | --- | --- | --- |
|  |  |  |  |  |

Describe the amount of frustration you experienced during the tutorial tasks:

| Low |  |  |  | High |
| --- | --- | --- | --- | --- |
|  |  |  |  |  |

Please describe thoughts and comments (if any) that you have about the tutorial section (related to individual tasks, overall structure, etc.):

| The only concern of suggestion I would make would be in regards to clarifying a co-matrix itself.  For example, it is not very clear when a box is shaded whether the shaded box represents   1. one specific tweet, or 2. a relationship that exists between two or more tweets.   Confusion can arise in terms of how many “degrees of separation” exist between the places or hashtags listed in the co-matrix that interest in a colored box.  Otherwise, great job at explaining in a very clear manner. ☺ |
| --- |

# Task 1 – Hashtags and Floods

Please enter your findings from **Part A** of this task in the box below:

| #theState: Gerry Melendez, a documentary photographer from Columbia, SC noted that there appear to be high water levels in the High Congaree River, specifically under the Gervais Street bridge.  #MoncksCorner: The Post and Courier, a news source based in and around Charleston, SC, noted that there was extreme flooding on Hwy 17 at the Wadboo Bridge, thus encouraging residents and visitors to avoid the roads. The scene is a dramatic video of high-level water rushing feet above the ground and through forests of trees.  #SCflooding: I-26 bridge over the Saluda river is drastically flooded, according to a picture from an eyewitness.  Etc. |
| --- |

Please enter your findings from **Part B** of this task in the box below:

| #FirstAlertWIS10: I-26 at the Saluda river bridge is closed due to the water/debris dangerously close to the top.  #chstrfc: From the Charleston police – the Main Road at Limestone bridge finally became over for motorists leaving Johns island, presumably after floodwaters had died down.  #sctweets: From personal eyewitnesses, the Congaree River at Blossom St. bridge is heavily flooded.  Etc. |
| --- |

# Task 2 – South Carolina Bridges

Please enter your findings from **Part A** of this task in the box below:

| Columbia: Gervais street bridge appears to be heavily flooded (an eyewitness gave pictures of before and after).  Gervais Street Bridge: Similar to information from above, another eyewitness shows pictures of different views of the flooded bridge. Even after an extended period of time, the flood waters appear to be moving swiftly. |
| --- |

Please enter your findings from **Part B** of this task in the box below:

| Charleston: Drivers near Bacon Bridge being detoured due to a slipping crane (as reported by @Live5News).  Congaree: High Congaree river water levels under the Gervais street bridge.  Wadboo Bridge: Heavy flooding near Hwy 17 below the Wadboo Bridge, the Post and Courier reports (many other repeat tweets). Once again, the video is graphic in the eyewitness depiction of rapidly rolling waters.  Etc. |
| --- |

# Joint Feedback for Tasks 1 and 2

Describe the level of mental demand for these tasks (e.g. amount of thinking, remembering, searching, etc.):

| Low |  |  |  | High |
| --- | --- | --- | --- | --- |
|  |  |  |  |  |

Describe the level of physical demand for these tasks (e.g. amount of clicking, scrolling, typing, etc.):

| Low |  |  |  | High |
| --- | --- | --- | --- | --- |
|  |  |  |  |  |

Describe the level of temporal demand for these tasks (i.e. the amount of time pressure you experienced):

| Low |  |  |  | High |
| --- | --- | --- | --- | --- |
|  |  |  |  |  |

Describe your level of performance for these tasks (i.e. how much success you think you had in accomplishing the goals of this task):

| Low |  |  |  | High |
| --- | --- | --- | --- | --- |
|  |  |  |  |  |

Describe the amount of effort you put into these tasks to achieve your level of performance:

| Low |  |  |  | High |
| --- | --- | --- | --- | --- |
|  |  |  |  |  |

Describe the amount of frustration you experienced during these tasks:

| Low |  |  |  | High |
| --- | --- | --- | --- | --- |
|  |  |  |  |  |

Describe specific ways, if any, in which individual tool features helped or hampered your progress in these tasks:

| I appreciated having a variety of “paths” available to explore a certain topic. My only concern would be if I was asked to do this myself, I might not know exactly what paths to investigate in order to be sure to not miss any information. I suppose that I would need more practice to be familiar enough with the program to cover all the data necessary without having specific instructions (such as for task 1 and task 2). |
| --- |

Please describe any additional thoughts that were not covered by the previous questions (including thoughts about SensePlace3, individual tasks, the study as a whole, etc.):

| Great tutorial about the potential of the co-matrix. Once again, I am a little concerned about the user interface for those who do not specific instructions (or who are unwilling to read detailed instructions given to them), but the capabilities (especially of the co-matrix) are great. ☺ |
| --- |

You are done! Check in with the scientist to receive your payment.
