# Supplementary material for: Augmenting geovisual analytics of social media data with heterogeneous information network mining—Cognitive plausibility assessment
Source: PLoS One. 2018 Dec 4;13(12):e0206906. doi: 10.1371/journal.pone.0206906 (PMC6279051; doi:10.1371/journal.pone.0206906)
Supplement: S3 File — This file contains, in a compressed format, the raw data provided by the participants of the study by means of the study questionnaire. (ZIP) [file pone.0206906.s003.zip › questionnaireResults/questionnaire.netw.3.docx]

# Tutorial Feedback

Describe the level of mental demand for the tutorial tasks (e.g. amount of thinking, remembering, searching, etc.):

| Low |  |  |  | High |
| --- | --- | --- | --- | --- |
|  |  |  |  |  |

Describe the level of physical demand for the tutorial tasks (e.g. amount of clicking, scrolling, typing, etc.):

| Low |  |  |  | High |
| --- | --- | --- | --- | --- |
|  |  |  |  |  |

Describe the level of temporal demand for the tutorial tasks (i.e. the amount of time pressure you experienced):

| Low |  |  |  | High |
| --- | --- | --- | --- | --- |
|  |  |  |  |  |

Describe your level of performance for the tutorial tasks (i.e. how much success you think you had in accomplishing the goals of this task):

| Low |  |  |  | High |
| --- | --- | --- | --- | --- |
|  |  |  |  |  |

Describe the amount of effort you put into the tutorial tasks to achieve your level of performance:

| Low |  |  |  | High |
| --- | --- | --- | --- | --- |
|  |  |  |  |  |

Describe the amount of frustration you experienced during the tutorial tasks:

| Low |  |  |  | High |
| --- | --- | --- | --- | --- |
|  |  |  |  |  |

Please describe thoughts and comments (if any) that you have about the tutorial section (related to individual tasks, overall structure, etc.):

| I thought that the tutorial section was very helpful in understanding what is going on behind the matrix. The way that it was explained was very logical and helpful; it made a lot of sense. The progression of the concepts was also executed very well. I now feel more like I know what the point of this program is and how to use it better in order to find relations among hashtags and places. Although some of the actual tasks themselves seemed a little bit obvious or simple, I think it was a great instructional tool. |
| --- |

# Task 1 – Hashtags and Floods

Please enter your findings from **Part A** of this task in the box below:

| #thestate: seems to be related to the Gervais Street bridge. These were also all tweets that had been a RT of the original tweet. This combination of hashtags is not as popular as it might appear to be.  #MoncksCorner: All tweets for this were also RT’s of the original tweet. The hashtag seems to also be related to a location near the Wadboo Bridge of Hwy. 17.  #SCflooding: This tweet was also RT’d and, thus, is less popular than appears. Seems to be related to the Saluda River and I-26. |
| --- |

Please enter your findings from **Part B** of this task in the box below:

| #FirstAlertWIS10: This hashtag is from a tweet by a news station showing footage of flooding. It was one tweet that was RT’d or RT’d with text.  #sctweets: This hashtag was used two different times in tweets from the same user, Gerry Mendelez. All other tweets that showed up in the matrix were RT’s of one of those original tweets.  #joaquin: This hashtag was also used just a couple of times mostly by news anchors’ twitter accounts. The rest of the tweets were RT’s of those original tweets with the hashtag. |
| --- |

# Task 2 – South Carolina Bridges

Please enter your findings from **Part A** of this task in the box below:

| Columbia; This is a city in South Carolina, the other location tag in these tweets.  Gervais Street Bridge; This is a bridge in Columbia, SC. |
| --- |

Please enter your findings from **Part B** of this task in the box below:

| Congaree; Congaree is a River that had flooding.  Wadboo Bridge; Wadboo Bridge is a bridge that was damaged in Moncks Corner.  Eastover; This seems to be a town in SC.  Bacon Bridge; This is a bridge that had live coverage from a news channel. |
| --- |

Please enter your findings from **Part C** of this task in the box below:

| I speculate that the findings from Part A were different from Part B in Task 2 because the first co-occurrence matrix was only looking at tweets that mentioned both locations. Part B, however, was searching for related locations through the use of a hashtag. This would create different results in the query because individual tweets do not always use more than one location tag. Using the hashtag as the basis for searching for related places is much more fruitful in its results. |
| --- |

# Joint Feedback for Tasks 1 and 2

Describe the level of mental demand for these tasks (e.g. amount of thinking, remembering, searching, etc.):

| Low |  |  |  | High |
| --- | --- | --- | --- | --- |
|  |  |  |  |  |

Describe the level of physical demand for these tasks (e.g. amount of clicking, scrolling, typing, etc.):

| Low |  |  |  | High |
| --- | --- | --- | --- | --- |
|  |  |  |  |  |

Describe the level of temporal demand for these tasks (i.e. the amount of time pressure you experienced):

| Low |  |  |  | High |
| --- | --- | --- | --- | --- |
|  |  |  |  |  |

Describe your level of performance for these tasks (i.e. how much success you think you had in accomplishing the goals of this task):

| Low |  |  |  | High |
| --- | --- | --- | --- | --- |
|  |  |  |  |  |

Describe the amount of effort you put into these tasks to achieve your level of performance:

| Low |  |  |  | High |
| --- | --- | --- | --- | --- |
|  |  |  |  |  |

Describe the amount of frustration you experienced during these tasks:

| Low |  |  |  | High |
| --- | --- | --- | --- | --- |
|  |  |  |  |  |

Describe specific ways, if any, in which individual tool features helped or hampered your progress in these tasks:

| The co-occurrence matrix was very easy to use, and I was able to line the tweet window up just to the side of the matrix so that I could see all of the windows in my workspace. The only thing that was a little difficult is the small text in the matrix. Sometimes the letters would be smooshed together. Also, I wish that when a box is selected, that the row and column tags would remain highlighted instead of just when the mouse is scrolled over it. Other than those few things, I found this to be very easy to use. |
| --- |

Please describe any additional thoughts that were not covered by the previous questions (including thoughts about SensePlace3, individual tasks, the study as a whole, etc.):

| In comparison to the last time I used this, I was much better at using the program. I attribute this to my increased understanding and knowledge of how the program works and how I can use it to find what I am looking for. |
| --- |

You are done! Check in with the scientist to receive your payment.
