# Supplementary material for: Augmenting geovisual analytics of social media data with heterogeneous information network mining—Cognitive plausibility assessment
Source: PLoS One. 2018 Dec 4;13(12):e0206906. doi: 10.1371/journal.pone.0206906 (PMC6279051; doi:10.1371/journal.pone.0206906)
Supplement: S3 File — This file contains, in a compressed format, the raw data provided by the participants of the study by means of the study questionnaire. (ZIP) [file pone.0206906.s003.zip › questionnaireResults/questionnaire.netw.4.docx]

# Tutorial Feedback

Describe the level of mental demand for the tutorial tasks (e.g. amount of thinking, remembering, searching, etc.):

| Low |  |  |  | High |
| --- | --- | --- | --- | --- |
|  |  |  |  |  |

Describe the level of physical demand for the tutorial tasks (e.g. amount of clicking, scrolling, typing, etc.):

| Low |  |  |  | High |
| --- | --- | --- | --- | --- |
|  |  |  |  |  |

Describe the level of temporal demand for the tutorial tasks (i.e. the amount of time pressure you experienced):

| Low |  |  |  | High |
| --- | --- | --- | --- | --- |
|  |  |  |  |  |

Describe your level of performance for the tutorial tasks (i.e. how much success you think you had in accomplishing the goals of this task):

| Low |  |  |  | High |
| --- | --- | --- | --- | --- |
|  |  |  |  |  |

Describe the amount of effort you put into the tutorial tasks to achieve your level of performance:

| Low |  |  |  | High |
| --- | --- | --- | --- | --- |
|  |  |  |  |  |

Describe the amount of frustration you experienced during the tutorial tasks:

| Low |  |  |  | High |
| --- | --- | --- | --- | --- |
|  |  |  |  |  |

Please describe thoughts and comments (if any) that you have about the tutorial section (related to individual tasks, overall structure, etc.):

| The tutorial section clearly explained the logic underlying the matrix. |
| --- |

# Task 1 – Hashtags and Floods

Please enter your findings from **Part A** of this task in the box below:

| The hashtags #the state, #Monckscorner, #flood, #scflooding, #columbiasc and #congateeriver seem most useful.  #the state: This hashtag offers government information.  #Monckscorner: This hashtag offers flood information, and often links to #the state.  #flood: This hashtag is on topic with flooding.  #scflooding: This hashtag is on topic with flooding.  #columbiasc: This hashtag provides detail about a place that flooded.  #congatee river: This hashtag connects to a river that is flooding. |
| --- |

Please enter your findings from **Part B** of this task in the box below:

| #FirstAlertsWIS10: This hashtag includes information about specific roads that are flooded out  #chstrfc: This hashtag provides information from law enforcement.  #sctweets: This hashtag provides information about flooding in specific places.  #WLTX19: This hashtag has links to photos of places that flooded.  #chsnews: This hashtag provides information about road conditions.  #project365: This hashtag provides links to images of flooded bridges.  #day274: This hashtag also provides links to images. |
| --- |

# Task 2 – South Carolina Bridges

Please enter your findings from **Part A** of this task in the box below:

| Charleston: a city that experienced flooding  Gervais Street Bridge: a bridge that flooded out |
| --- |

Please enter your findings from **Part B** of this task in the box below:

| Bacon Bridge: a bridge that was flooded  Black River: a river that flooded  Brown’s Ferry Bridge: a bridge along the Black River that flooded  Cannon Bridge: along the flood river  Cayce: area closed due to flooding  Columbia: city affected by flooding  Congratee: flooded river  Eastover: flooded area  Georgetown: flooded county  Limehouse Bridge: closed bridge  Saluda River: high water  SC: state name  Wadboo Bridge: flooded bridge  West Columbia: area of the flooded city |
| --- |

Please enter your findings from **Part C** of this task in the box below:

| Part B generated more hits because it made hashtags the center of its search. People use hashtags when they tweet, so a search centered on hashtags is going to gather better results than a search that is centered on the contents of the tweets. |
| --- |

# Joint Feedback for Tasks 1 and 2

Describe the level of mental demand for these tasks (e.g. amount of thinking, remembering, searching, etc.):

| Low |  |  |  | High |
| --- | --- | --- | --- | --- |
|  |  |  |  |  |

Describe the level of physical demand for these tasks (e.g. amount of clicking, scrolling, typing, etc.):

| Low |  |  |  | High |
| --- | --- | --- | --- | --- |
|  |  |  |  |  |

Describe the level of temporal demand for these tasks (i.e. the amount of time pressure you experienced):

| Low |  |  |  | High |
| --- | --- | --- | --- | --- |
|  |  |  |  |  |

Describe your level of performance for these tasks (i.e. how much success you think you had in accomplishing the goals of this task):

| Low |  |  |  | High |
| --- | --- | --- | --- | --- |
|  |  |  |  |  |

Describe the amount of effort you put into these tasks to achieve your level of performance:

| Low |  |  |  | High |
| --- | --- | --- | --- | --- |
|  |  |  |  |  |

Describe the amount of frustration you experienced during these tasks:

| Low |  |  |  | High |
| --- | --- | --- | --- | --- |
|  |  |  |  |  |

Describe specific ways, if any, in which individual tool features helped or hampered your progress in these tasks:

| The tools were easy to use. The matrix is particularly easy to use because it can be sorted in multiple ways and the colors indicate the strength of the connection between the eleemnts. |
| --- |

Please describe any additional thoughts that were not covered by the previous questions (including thoughts about SensePlace3, individual tasks, the study as a whole, etc.):

| This set of tasks was easier than the previous set of tasks. |
| --- |

You are done! Check in with the scientist to receive your payment.
