# Supplementary material for: Augmenting geovisual analytics of social media data with heterogeneous information network mining—Cognitive plausibility assessment
Source: PLoS One. 2018 Dec 4;13(12):e0206906. doi: 10.1371/journal.pone.0206906 (PMC6279051; doi:10.1371/journal.pone.0206906)
Supplement: S3 File — This file contains, in a compressed format, the raw data provided by the participants of the study by means of the study questionnaire. (ZIP) [file pone.0206906.s003.zip › questionnaireResults/questionnaire.netw.5.docx]

# Tutorial Feedback

Describe the level of mental demand for the tutorial tasks (e.g. amount of thinking, remembering, searching, etc.):

| Low |  |  |  | High |
| --- | --- | --- | --- | --- |
|  |  |  |  |  |

Describe the level of physical demand for the tutorial tasks (e.g. amount of clicking, scrolling, typing, etc.):

| Low |  |  |  | High |
| --- | --- | --- | --- | --- |
|  |  |  |  |  |

Describe the level of temporal demand for the tutorial tasks (i.e. the amount of time pressure you experienced):

| Low |  |  |  | High |
| --- | --- | --- | --- | --- |
|  |  |  |  |  |

Describe your level of performance for the tutorial tasks (i.e. how much success you think you had in accomplishing the goals of this task):

| Low |  |  |  | High |
| --- | --- | --- | --- | --- |
|  |  |  |  |  |

Describe the amount of effort you put into the tutorial tasks to achieve your level of performance:

| Low |  |  |  | High |
| --- | --- | --- | --- | --- |
|  |  |  |  |  |

Describe the amount of frustration you experienced during the tutorial tasks:

| Low |  |  |  | High |
| --- | --- | --- | --- | --- |
|  |  |  |  |  |

Please describe thoughts and comments (if any) that you have about the tutorial section (related to individual tasks, overall structure, etc.):

| Very patient. Nice! |
| --- |

# Task 1 – Hashtags and Floods

Please enter your findings from **Part A** of this task in the box below:

| Hashtags:  # chsnews – Charleston news;  # thestate – the state news;  # MoncksCorner – a company named by a place?  # flood – flood event  # Orangeburg – place  # Bamberg – place  # joaquin – people  # SCflooding – flood event  # columbiasc—place  # congareeriver – a river, place |
| --- |

Please enter your findings from **Part B** of this task in the box below:

| Hashtags:  # FirstAlertTWIS10 – news  # chstrfc – Charleston traffic  # sctweets – south Carolina news  # WLTX19 – tv channel  # WLTXtraffic – traffic channel  # project365 -- ?  #day274 -- ?  # jobs – job info  # jobfairusa – job info  # careerbuilder – job info  # SCWX – news  # ColumbiaFlood – event  # charlestonflooding – event  # SC – place |
| --- |

# Task 2 – South Carolina Bridges

Please enter your findings from **Part A** of this task in the box below:

| Columbia – city  Gervais street bridge – bridge |
| --- |

Please enter your findings from **Part B** of this task in the box below:

| Bacon Bridge – bridge;  Black River – river;  BrownsFerryBridge – bridge;  Cannon Bridge – bridge;  Cayce – place?  Charleston – place;  Congaree – river name/ place;  Eastover – place  Georgetown – place;  Limehouse bridge – bridge;  Saluda River – river;  SC – south Carolina  Wadboo bridge – bridge;  West Columbia – place |
| --- |

Please enter your findings from **Part C** of this task in the box below:

| 1. The results in part A only includes tweets mentioned two locations at the same time. While part B also include locations mentioned in different tweets which are connected by the same hashtag. |
| --- |

# Joint Feedback for Tasks 1 and 2

Describe the level of mental demand for these tasks (e.g. amount of thinking, remembering, searching, etc.):

| Low |  |  |  | High |
| --- | --- | --- | --- | --- |
|  |  |  |  |  |

Describe the level of physical demand for these tasks (e.g. amount of clicking, scrolling, typing, etc.):

| Low |  |  |  | High |
| --- | --- | --- | --- | --- |
|  |  |  |  |  |

Describe the level of temporal demand for these tasks (i.e. the amount of time pressure you experienced):

| Low |  |  |  | High |
| --- | --- | --- | --- | --- |
|  |  |  |  |  |

Describe your level of performance for these tasks (i.e. how much success you think you had in accomplishing the goals of this task):

| Low |  |  |  | High |
| --- | --- | --- | --- | --- |
|  |  |  |  |  |

Describe the amount of effort you put into these tasks to achieve your level of performance:

| Low |  |  |  | High |
| --- | --- | --- | --- | --- |
|  |  |  |  |  |

Describe the amount of frustration you experienced during these tasks:

| Low |  |  |  | High |
| --- | --- | --- | --- | --- |
|  |  |  |  |  |

Describe specific ways, if any, in which individual tool features helped or hampered your progress in these tasks:

| 1. The placement of the words on the top is hard to recognize, especially considering some of hashtags are meaningless. So sometimes I have to click on the tweet to figure out what are those words. 2. I like when I click on one cell, both of the axis are highlighted, which made it easier to identify. 3. The number only indicates total number of tweets. It would be good it the number of cells can also be displayed. For double check purpose, I have to count the number of cells myself. |
| --- |

Please describe any additional thoughts that were not covered by the previous questions (including thoughts about SensePlace3, individual tasks, the study as a whole, etc.):

| I find myself didn’t use the map. Not very clear about the function of this map. Also another question. For the two models in task2, not sure if the results of Part A will definitely be a subset of Part B. From the model, it seems not. But I feel it should be. |
| --- |

You are done! Check in with the scientist to receive your payment.
