# Supplementary material for: Augmenting geovisual analytics of social media data with heterogeneous information network mining—Cognitive plausibility assessment
Source: PLoS One. 2018 Dec 4;13(12):e0206906. doi: 10.1371/journal.pone.0206906 (PMC6279051; doi:10.1371/journal.pone.0206906)
Supplement: S3 File — This file contains, in a compressed format, the raw data provided by the participants of the study by means of the study questionnaire. (ZIP) [file pone.0206906.s003.zip › questionnaireResults/questionnaire.netw.6.docx]

# Tutorial Feedback

Describe the level of mental demand for the tutorial tasks (e.g. amount of thinking, remembering, searching, etc.):

| Low |  |  |  | High |
| --- | --- | --- | --- | --- |
|  |  |  |  |  |

Describe the level of physical demand for the tutorial tasks (e.g. amount of clicking, scrolling, typing, etc.):

| Low |  |  |  | High |
| --- | --- | --- | --- | --- |
|  |  |  |  |  |

Describe the level of temporal demand for the tutorial tasks (i.e. the amount of time pressure you experienced):

| Low |  |  |  | High |
| --- | --- | --- | --- | --- |
|  |  |  |  |  |

Describe your level of performance for the tutorial tasks (i.e. how much success you think you had in accomplishing the goals of this task):

| Low |  |  |  | High |
| --- | --- | --- | --- | --- |
|  |  |  |  |  |

Describe the amount of effort you put into the tutorial tasks to achieve your level of performance:

| Low |  |  |  | High |
| --- | --- | --- | --- | --- |
|  |  |  |  |  |

Describe the amount of frustration you experienced during the tutorial tasks:

| Low |  |  |  | High |
| --- | --- | --- | --- | --- |
|  |  |  |  |  |

Please describe thoughts and comments (if any) that you have about the tutorial section (related to individual tasks, overall structure, etc.):

| It provides a good overview for user to gain an understanding of how the tool works at a basic level. |
| --- |

# Task 1 – Hashtags and Floods

Please enter your findings from **Part A** of this task in the box below:

| #Chsnews – The local news service in the affected area  #thestate – associated with high river level at the Gervais Street Bridge  #MonckCorner – An area heavily affected by the flood  #flood – alternative hashtag used for tweets about the flooding  #Orangeburg - An area affected by the flood  #Bamburg - An area affected by the flood  #joaquin – the weather system associated with the flood  #SCflooding - alternative hashtag used for tweets about the flooding  #columbiasc - An area affected by the flood  #congareeriver - An area affected by the flood |
| --- |

Please enter your findings from **Part B** of this task in the box below:

| #FirstAlertWIS10 - local news service in the affected area  #chstrfc – hashtag used for traffic information for the Charleston area  #sctweets - hashtag used for tweets about/from SC  #WLTX19 - local news service in the affected area  #WLTXtraffic - hashtag used for traffic information by a local news service in the affected area  #project365 – unknown/no relation  #day274 – unknown/no relation  #jobs – unknown/no relation  #jobfairuse - unknown/no relation  #careerbuilder - unknown/no relation  #SCWX - hashtag used for tweets about weather in SC  #columbiaflood – tweets abt flooding in the Columbia area  #charlestonflooding - tweets abt flooding in the Charleston area  #SC – hashtag used for tweets about/from SC |
| --- |

# Task 2 – South Carolina Bridges

Please enter your findings from **Part A** of this task in the box below:

| Columbia – A City in South Carolina  Gervais street bridge – a bridge in Columbia, South Carolina |
| --- |

Please enter your findings from **Part B** of this task in the box below:

| Bacon Bridge – A bridge in SC  Black River – A river in SC  Browns Ferry Bridge – A bridge in SC  Cannon Bridge – A bridge in SC  Cayce – A place in SC  Charleston – City in SC  Congaree – River in SC  Eastover – a place in SC  Georgetown – a county in SC  Limehouse Bridge – a bridge in SC  Saluda River – a river in SC  SC – abbreviation for South Carolina  Wadboo Bridge – a bridge in Moncks Corner, SC  West Columbia – an area of the City of Columbia in SC |
| --- |

Please enter your findings from **Part C** of this task in the box below:

| Part A identifies Tweets that mentions both locations in the same tweet, thus providing a more direct relation of the locations. Meanwhile, Part B links locations of tweets that use the same hashtag, thus providing a more general relation between the locations. |
| --- |

# Joint Feedback for Tasks 1 and 2

Describe the level of mental demand for these tasks (e.g. amount of thinking, remembering, searching, etc.):

| Low |  |  |  | High |
| --- | --- | --- | --- | --- |
|  |  |  |  |  |

Describe the level of physical demand for these tasks (e.g. amount of clicking, scrolling, typing, etc.):

| Low |  |  |  | High |
| --- | --- | --- | --- | --- |
|  |  |  |  |  |

Describe the level of temporal demand for these tasks (i.e. the amount of time pressure you experienced):

| Low |  |  |  | High |
| --- | --- | --- | --- | --- |
|  |  |  |  |  |

Describe your level of performance for these tasks (i.e. how much success you think you had in accomplishing the goals of this task):

| Low |  |  |  | High |
| --- | --- | --- | --- | --- |
|  |  |  |  |  |

Describe the amount of effort you put into these tasks to achieve your level of performance:

| Low |  |  |  | High |
| --- | --- | --- | --- | --- |
|  |  |  |  |  |

Describe the amount of frustration you experienced during these tasks:

| Low |  |  |  | High |
| --- | --- | --- | --- | --- |
|  |  |  |  |  |

Describe specific ways, if any, in which individual tool features helped or hampered your progress in these tasks:

| It would be better if the co-matrix window could itself be integrated into the main senseplace application. This would negate the need to switch back and forth between windows and may improve user performance. Many of the tweets shown in the application were no longer up or accessible. |
| --- |

Please describe any additional thoughts that were not covered by the previous questions (including thoughts about SensePlace3, individual tasks, the study as a whole, etc.):

| The tool is great for helping discern patterns and relation. Although it clearly still resulted in some false positive when relation tweet using a more general relation (Task 1B). |
| --- |

You are done! Check in with the scientist to receive your payment.
