# Supplementary material for: Augmenting geovisual analytics of social media data with heterogeneous information network mining—Cognitive plausibility assessment
Source: PLoS One. 2018 Dec 4;13(12):e0206906. doi: 10.1371/journal.pone.0206906 (PMC6279051; doi:10.1371/journal.pone.0206906)
Supplement: S3 File — This file contains, in a compressed format, the raw data provided by the participants of the study by means of the study questionnaire. (ZIP) [file pone.0206906.s003.zip › questionnaireResults/questionnaire.netw.7.docx]

# Tutorial Feedback

Describe the level of mental demand for the tutorial tasks (e.g. amount of thinking, remembering, searching, etc.):

| Low |  |  |  | High |
| --- | --- | --- | --- | --- |
|  |  |  |  |  |

Describe the level of physical demand for the tutorial tasks (e.g. amount of clicking, scrolling, typing, etc.):

| Low |  |  |  | High |
| --- | --- | --- | --- | --- |
|  |  |  |  |  |

Describe the level of temporal demand for the tutorial tasks (i.e. the amount of time pressure you experienced):

| Low |  |  |  | High |
| --- | --- | --- | --- | --- |
|  |  |  |  |  |

Describe your level of performance for the tutorial tasks (i.e. how much success you think you had in accomplishing the goals of this task):

| Low |  |  |  | High |
| --- | --- | --- | --- | --- |
|  |  |  |  |  |

Describe the amount of effort you put into the tutorial tasks to achieve your level of performance:

| Low |  |  |  | High |
| --- | --- | --- | --- | --- |
|  |  |  |  |  |

Describe the amount of frustration you experienced during the tutorial tasks:

| Low |  |  |  | High |
| --- | --- | --- | --- | --- |
|  |  |  |  |  |

Please describe thoughts and comments (if any) that you have about the tutorial section (related to individual tasks, overall structure, etc.):

| It was simple, easy, and intuitive especially with diagrams. |
| --- |

# Task 1 – Hashtags and Floods

Please enter your findings from **Part A** of this task in the box below:

| #chsnews – Limehouse Bridge closed via Charleston police.  #thestate – High Congaree River water levels under the Gervais Street bridge.  #MoncksCorner – People need to stay off Hwy. 17 at the Wadboo Bridge.  #flood – Gervais street bridge may have gone through flood.  #Orangeburg – South Edisto River bridge on Cannon Bridge suffers from flood.  #Bamburg – So, there is flood at the Orangeburg-Bamberg county line.  #joaquin - Gervais street bridge has gone through flood.  #SCflooding – I-26 Bridge over the Saluda River is flooding.  #columbiasc – The river near Gervais Street Bridge is flooding.  #congateeriver – Congatee River is flooding. |
| --- |

Please enter your findings from **Part B** of this task in the box below:

| #FirstAlertWIS10 Saluda River bridge closed due to water/debris dangerously close to top.  #chstric Main Rd at Limehouse Bridge is now open for motorists leaving Johns Island.  #sctweets Congaree River at Blossom St. bridge is flooding.  #WLTX19 High Congaree River water levels under the Gervais Street bridge.  #WLTXtraffic I-20 still closed at Broad River Bridge & I-26 closed at Saluda River.  #project365 Old railroad bridge over the Saluda River is flooding.  #day274 I-26 Bridge over the Saluda River is flooding.  #jobs advertisement for jobs (because of the use of the word ‘bridge’)  #jobfairusa advertisement for jobs (because of the use of the word ‘bridge’)  #careerbuilder advertisement for jobs (because of the use of the word ‘bridge’)  #SCWX SCDOT reporting 269 road closures, 143 bridge closures in South Carolina.  #ColumbiaFlood Roads are closed for flood.  #charlestonflooding Roads in Charleston may be closed for flood.  #SC South Carolina DOT receives $5 million in FHWA emergency relief funds for road and bridge damage.  #chsnews #flood #joaquin #columbiasc #congareeriver #SCflooding #thestate |
| --- |

# Task 2 – South Carolina Bridges

Please enter your findings from **Part A** of this task in the box below:

| Columbia - Columbia, South Carolina  Gervais street bridge - Gervais Street Bridge in Columbia, South Carolina   - Both suffer from flood. |
| --- |

Please enter your findings from **Part B** of this task in the box below:

| Bacon Bridge - Near Bacon Bridge in Charleston, drivers are being detoured due to a slipping crane.  Black River - Sabrina Johnson house on Black River just up from Browns Ferry Bridge in Georgetown county.  Browns Ferry Bridge – the same as above.  Cannon Bridge - South Edisto River bridge on Cannon Bridge Rd at the Orangeburg-Bamberg county line.  Cayce - 12th St. Ext. in Cayce is still closed below I-77.  Charleston - Limehouse Bridge closed via Charleston police.  Congaree - High Congaree River water levels under the Gervais Street bridge.  eastover - Ziegler road bridge in eastover is messy for flood.  Georgetown - Sabrina Johnson house on Black River just up from Browns Ferry Bridge in Georgetown cnty.  Limehouse Bridge - Limehouse Bridge closed via Charleston police.  Saluda River - I-26 Bridge over the Saluda River is overflowing.  sc - road out on the bridge on Zeigler Road in Eastover, sc.  Wadboo Bridge - Hwy. 17 at the Wadboo Bridge in MoncksCorner is broken.  West Columbia –Downtown Columbia on the left, West Columbia on the right both suffer from flood from Gervais St Bridge. |
| --- |

Please enter your findings from **Part C** of this task in the box below:

| 1. While part A looks at tweets mentioning multiple places, it is not very common in tweets. So, it results in fewer co-occurrences of different places. In part B, tweets are linked by the same hashtags, which allows inferring more associations among places. |
| --- |

# Joint Feedback for Tasks 1 and 2

Describe the level of mental demand for these tasks (e.g. amount of thinking, remembering, searching, etc.):

| Low |  |  |  | High |
| --- | --- | --- | --- | --- |
|  |  |  |  |  |

Describe the level of physical demand for these tasks (e.g. amount of clicking, scrolling, typing, etc.):

| Low |  |  |  | High |
| --- | --- | --- | --- | --- |
|  |  |  |  |  |

Describe the level of temporal demand for these tasks (i.e. the amount of time pressure you experienced):

| Low |  |  |  | High |
| --- | --- | --- | --- | --- |
|  |  |  |  |  |

Describe your level of performance for these tasks (i.e. how much success you think you had in accomplishing the goals of this task):

| Low |  |  |  | High |
| --- | --- | --- | --- | --- |
|  |  |  |  |  |

Describe the amount of effort you put into these tasks to achieve your level of performance:

| Low |  |  |  | High |
| --- | --- | --- | --- | --- |
|  |  |  |  |  |

Describe the amount of frustration you experienced during these tasks:

| Low |  |  |  | High |
| --- | --- | --- | --- | --- |
|  |  |  |  |  |

Describe specific ways, if any, in which individual tool features helped or hampered your progress in these tasks:

| In task 1, there were some irrelevant hashtags to flood (e.g. #jobs). It will be important to filter such hashtags.  When I tried to use the “network path” tool, it was not clear how I can make multiple links based on possible options (e.g. “about location” and so on). It would be good to show the inactive interface even though the second parameter has not been used. |
| --- |

Please describe any additional thoughts that were not covered by the previous questions (including thoughts about SensePlace3, individual tasks, the study as a whole, etc.):

|  |
| --- |

You are done! Check in with the scientist to receive your payment.
