# Supplementary material for: Augmenting geovisual analytics of social media data with heterogeneous information network mining—Cognitive plausibility assessment
Source: PLoS One. 2018 Dec 4;13(12):e0206906. doi: 10.1371/journal.pone.0206906 (PMC6279051; doi:10.1371/journal.pone.0206906)
Supplement: S3 File — This file contains, in a compressed format, the raw data provided by the participants of the study by means of the study questionnaire. (ZIP) [file pone.0206906.s003.zip › questionnaireResults/questionnaire.netw.8.docx]

# Tutorial Feedback

Describe the level of mental demand for the tutorial tasks (e.g. amount of thinking, remembering, searching, etc.):

| Low |  |  |  | High |
| --- | --- | --- | --- | --- |
|  |  |  |  |  |

Describe the level of physical demand for the tutorial tasks (e.g. amount of clicking, scrolling, typing, etc.):

| Low |  |  |  | High |
| --- | --- | --- | --- | --- |
|  |  |  |  |  |

Describe the level of temporal demand for the tutorial tasks (i.e. the amount of time pressure you experienced):

| Low |  |  |  | High |
| --- | --- | --- | --- | --- |
|  |  |  |  |  |

Describe your level of performance for the tutorial tasks (i.e. how much success you think you had in accomplishing the goals of this task):

| Low |  |  |  | High |
| --- | --- | --- | --- | --- |
|  |  |  |  |  |

Describe the amount of effort you put into the tutorial tasks to achieve your level of performance:

| Low |  |  |  | High |
| --- | --- | --- | --- | --- |
|  |  |  |  |  |

Describe the amount of frustration you experienced during the tutorial tasks:

| Low |  |  |  | High |
| --- | --- | --- | --- | --- |
|  |  |  |  |  |

Please describe thoughts and comments (if any) that you have about the tutorial section (related to individual tasks, overall structure, etc.):

| Pretty basic common sense but its good to go over to make sure everyone in the study has the same baseline knowledge on the topic. |
| --- |

# Task 1 – Hashtags and Floods

Please enter your findings from **Part A** of this task in the box below:

| Some hashtags I found:   - The state: All retweets of the same original tweet stating that there is high water levels under a bridge - #MoncksCorner: Tweets describing a road submerged in flooding. - #SCflooding: similar to SCFlood just using a present voice |
| --- |

Please enter your findings from **Part B** of this task in the box below:

| #FirstAlertWIS10 is compiled of mostly retweets discussing how a bridge has been closed due to the flooding.  #joaquin as above, all retweets of the same original tweet, showing what the bridge looked like before it was flooded.  #chstrfc a lot of different tweets talking about the current conditions of a lot of different bridges |
| --- |

# Task 2 – South Carolina Bridges

Please enter your findings from **Part A** of this task in the box below:

| Columbia: The Geravis Street Bridge is a topic of conversation among two tweeters who discuss how the water is affecting the bridge.  Gervais Street Bridge: The same tweets from the same people as above, just mentioned where the bridge was above. |
| --- |

Please enter your findings from **Part B** of this task in the box below:

| Bacon Bridge: A crane slipped and now drivers are being detoured from this location.  Black River: The tweet is about someone’s house who is either damaged or gone thanks to flooding in the Black River  Charleston: Tweets related to drivers being detoured because of the Bacon Bridge crash.  Congaree: Where the Geravis Street Bridge is located, all retweets about high water levels.  Other places include: Cannon Bridge, Cayce, Limehouse Bridge, Saluda River, and Wadboo Bridge. |
| --- |

Please enter your findings from **Part C** of this task in the box below:

| Part A only looks at one tweet that mentions two locations and relates them together that way, while Part B relates tweets that have shared the same hashtag and then relates the locations, providing a lot more details and matches than Part A. |
| --- |

# Joint Feedback for Tasks 1 and 2

Describe the level of mental demand for these tasks (e.g. amount of thinking, remembering, searching, etc.):

| Low |  |  |  | High |
| --- | --- | --- | --- | --- |
|  |  |  |  |  |

Describe the level of physical demand for these tasks (e.g. amount of clicking, scrolling, typing, etc.):

| Low |  |  |  | High |
| --- | --- | --- | --- | --- |
|  |  |  |  |  |

Describe the level of temporal demand for these tasks (i.e. the amount of time pressure you experienced):

| Low |  |  |  | High |
| --- | --- | --- | --- | --- |
|  |  |  |  |  |

Describe your level of performance for these tasks (i.e. how much success you think you had in accomplishing the goals of this task):

| Low |  |  |  | High |
| --- | --- | --- | --- | --- |
|  |  |  |  |  |

Describe the amount of effort you put into these tasks to achieve your level of performance:

| Low |  |  |  | High |
| --- | --- | --- | --- | --- |
|  |  |  |  |  |

Describe the amount of frustration you experienced during these tasks:

| Low |  |  |  | High |
| --- | --- | --- | --- | --- |
|  |  |  |  |  |

Describe specific ways, if any, in which individual tool features helped or hampered your progress in these tasks:

| Its good that the tool is set up how you would make the diagram, otherwise that would get confusing. |
| --- |

Please describe any additional thoughts that were not covered by the previous questions (including thoughts about SensePlace3, individual tasks, the study as a whole, etc.):

| When you describe using the path tool in the instructions, maybe provide more thorough instructions. I was confused when it appeared it wanted me to select an answer with multiple options, without knowing that once I clicked the first one, I would be able to select more after. |
| --- |

You are done! Check in with the scientist to receive your payment.
