# Supplementary material for: Augmenting geovisual analytics of social media data with heterogeneous information network mining—Cognitive plausibility assessment
Source: PLoS One. 2018 Dec 4;13(12):e0206906. doi: 10.1371/journal.pone.0206906 (PMC6279051; doi:10.1371/journal.pone.0206906)
Supplement: S3 File — This file contains, in a compressed format, the raw data provided by the participants of the study by means of the study questionnaire. (ZIP) [file pone.0206906.s003.zip › questionnaireResults/questionnaire.netw.9.docx]

# Tutorial Feedback

Describe the level of mental demand for the tutorial tasks (e.g. amount of thinking, remembering, searching, etc.):

| Low |  |  |  | High |
| --- | --- | --- | --- | --- |
|  |  |  |  |  |

Describe the level of physical demand for the tutorial tasks (e.g. amount of clicking, scrolling, typing, etc.):

| Low |  |  |  | High |
| --- | --- | --- | --- | --- |
|  |  |  |  |  |

Describe the level of temporal demand for the tutorial tasks (i.e. the amount of time pressure you experienced):

| Low |  |  |  | High |
| --- | --- | --- | --- | --- |
|  |  |  |  |  |

Describe your level of performance for the tutorial tasks (i.e. how much success you think you had in accomplishing the goals of this task):

| Low |  |  |  | High |
| --- | --- | --- | --- | --- |
|  |  |  |  |  |

Describe the amount of effort you put into the tutorial tasks to achieve your level of performance:

| Low |  |  |  | High |
| --- | --- | --- | --- | --- |
|  |  |  |  |  |

Describe the amount of frustration you experienced during the tutorial tasks:

| Low |  |  |  | High |
| --- | --- | --- | --- | --- |
|  |  |  |  |  |

Please describe thoughts and comments (if any) that you have about the tutorial section (related to individual tasks, overall structure, etc.):

| It is good. The instructions are very clear, and the diagrams are easy to understand. The overall structure is reasonable, starting with easy tasks and then goes to more complicated ones. |
| --- |

# Task 1 – Hashtags and Floods

Please enter your findings from **Part A** of this task in the box below:

| #chsnews This hashtag should be the name of a media reporting about the flood  #theState It refers to ‘The State Newspaper’  #MoncksCorner It is a place in SC related to the flood.  #flood It means flood…  #Orangeburg It is a place in SC related to the flood.  #Bamberg It is a place in SC related to the flood.  #joaquin It is the hurricane caused the flood.  #SCflooding It is similar to #SCflood, referring to that SC is flooding.  #columbiasc A place in SC.  #congareeriver It refers to the Congaree river in SC. |
| --- |

Please enter your findings from **Part B** of this task in the box below:

| #FirstAlertWIS10 Weather alert in WIS tv channel 10.  #chstrfc maybe a traffic tool of chs  #sctweets Tweets in SC.  #WLTX19 A tv news channel  #WLTXtraffic maybe a traffic tool from WLTX  #project365 an app or a project to keep journals of everyday  #day274 The 274 day of the project 365.  #jobs just means jobs…  #jobfairusa a twitter account named Job Fair USA.  #careerbuilder just means career builder…  #SCWX SC weather forecast?  #ColumbiaFlood flood in Columbia in SC  #charlestonflooding Charleston in SC is flooding.  #SC just means SC… |
| --- |

# Task 2 – South Carolina Bridges

Please enter your findings from **Part A** of this task in the box below:

| Gervais street bridge (Gervais Street Bridge is a historic bridge in South Carolina)  Columbia (Columbia is the capital of and largest city in the U.S. state of South Carolina) |
| --- |

Please enter your findings from **Part B** of this task in the box below:

| Congaree (The Congaree River is a short but wide river in South Carolina)  Wadboo Bridge (Bridge in South Carolina)  Charleston (A South Carolina city founded in 1670)  Saluda River (The Saluda River is a principal tributary of the Congaree River in SC)  Eastover (Eastover is a town in Richland County, South Carolina)  Bacon Bridge (A bridge in Summerville, SC)  Limehouse Bridge (The John F. Limehouse Memorial Bridge in SC)  sc (South Carolina)  West Columbia (A city in the suburban eastern sections of Lexington County, South Carolina)  Cayce (Cayce is a city in Lexington and Richland counties in the U.S. state of South Carolina)  Black River (The Black River is a 151-mile-long blackwater river in South Carolina in the United States)  Browns Ferry Bridge (A bridge in Georgetown, SC)  Cape Fear River (The Cape Fear River is a 202 miles long blackwater river in east central North Carolina) |
| --- |

Please enter your findings from **Part C** of this task in the box below:

| They are different because in Part A, it only shows the places mentioned in one tweet, while usually a tweet is about the things happened in one place. In part B, it shows the places related to the same event (represented by the hashtag.) Since an event can spread across many places, we could find more places in Part B. |
| --- |

# Joint Feedback for Tasks 1 and 2

Describe the level of mental demand for these tasks (e.g. amount of thinking, remembering, searching, etc.):

| Low |  |  |  | High |
| --- | --- | --- | --- | --- |
|  |  |  |  |  |

Describe the level of physical demand for these tasks (e.g. amount of clicking, scrolling, typing, etc.):

| Low |  |  |  | High |
| --- | --- | --- | --- | --- |
|  |  |  |  |  |

Describe the level of temporal demand for these tasks (i.e. the amount of time pressure you experienced):

| Low |  |  |  | High |
| --- | --- | --- | --- | --- |
|  |  |  |  |  |

Describe your level of performance for these tasks (i.e. how much success you think you had in accomplishing the goals of this task):

| Low |  |  |  | High |
| --- | --- | --- | --- | --- |
|  |  |  |  |  |

Describe the amount of effort you put into these tasks to achieve your level of performance:

| Low |  |  |  | High |
| --- | --- | --- | --- | --- |
|  |  |  |  |  |

Describe the amount of frustration you experienced during these tasks:

| Low |  |  |  | High |
| --- | --- | --- | --- | --- |
|  |  |  |  |  |

Describe specific ways, if any, in which individual tool features helped or hampered your progress in these tasks:

| For the tweets in the left column of the main window, it seems that if the tweet is related to several hashtags/places, it would not pop up to the top when I click on the second hashtag/place in the CoMatrix.  For the CoMatrix, it would be better that I can manually change a little bit about the ordering. Since SC is not the top row/column, the related words would not concentrate in the top-left part. So I might miss some of them if I do not pay enough attention. |
| --- |

Please describe any additional thoughts that were not covered by the previous questions (including thoughts about SensePlace3, individual tasks, the study as a whole, etc.):

|  |
| --- |

You are done! Check in with the scientist to receive your payment.
